# New Insights on Genetic Diagnostics in Cardiomyopathy and Arrhythmia Patients Gained by Stepwise Exome Data Analysis

**DOI:** 10.3390/jcm9072168

**Published:** 2020-07-09

**Authors:** Konstantinos Kolokotronis, Natalie Pluta, Eva Klopocki, Erdmute Kunstmann, Daniel Messroghli, Christoph Maack, Shai Tejman-Yarden, Michael Arad, Simone Rost, Brenda Gerull

**Affiliations:** 1 Institute of Human Genetics, Biocenter, Julius-Maximilians-University Würzburg, 97074 Würzburg, Germany; konstantinos.kolokotronis@uni-wuerzburg.de (K.K.); natalie.pluta@uni-wuerzburg.de (N.P.); eva.klopocki@uni-wuerzburg.de (E.K.); kunstmann@biozentrum.uni-wuerzburg.de (E.K.); simone.rost@biozentrum.uni-wuerzburg.de (S.R.); 2 German Heart Center Berlin, Department of Internal Medicine-Cardiology, 13353 Berlin, Germany; daniel.messroghli@charite.de; 3 Department of Translational Research, Comprehensive Heart Failure Center (CHFC), University Clinic Würzburg, 97078 Würzburg, Germany; Maack_C@ukw.de; 4 The Safra International Congenital Heart Center. Sheba Medical Center and Sackler School of Medicine, Tel Aviv University, Ramat Gan 5365601, Israel; Shai.TejmanYarden@sheba.health.gov.il; 5 Heart Failure Institute and Leviev Heart Center, Sheba Medical Center and Sackler School of Medicine, Tel Aviv University, Ramat Gan 5365601, Israel; michael.arad@sheba.health.gov.il; 6 Department of Cardiovascular Genetics, Comprehensive Heart Failure Center (CHFC) and Department of Medicine I, University Clinic Würzburg, 97078 Würzburg, Germany

**Keywords:** cardiomyopathy, cardiogenetics, whole exome sequencing, targeted gene panel, candidate genes

## Abstract

Inherited cardiomyopathies are characterized by clinical and genetic heterogeneity that challenge genetic diagnostics. In this study, we examined the diagnostic benefit of exome data compared to targeted gene panel analyses, and we propose new candidate genes. We performed exome sequencing in a cohort of 61 consecutive patients with a diagnosis of cardiomyopathy or primary arrhythmia, and we analyzed the data following a stepwise approach. Overall, in 64% of patients, a variant of interest (VOI) was detected. The detection rate in the main sub-cohort consisting of patients with dilated cardiomyopathy (DCM) was much higher than previously reported (25/36; 69%). The majority of VOIs were found in disease-specific panels, while a further analysis of an extended panel and exome data led to an additional diagnostic yield of 13% and 5%, respectively. Exome data analysis also detected variants in candidate genes whose functional profile suggested a probable pathogenetic role, the strongest candidate being a truncating variant in *STK38.* In conclusion, although the diagnostic yield of gene panels is acceptable for routine diagnostics, the genetic heterogeneity of cardiomyopathies and the presence of still-unknown causes favor exome sequencing, which enables the detection of interesting phenotype–genotype correlations, as well as the identification of novel candidate genes.

## 1. Introduction

Inherited cardiomyopathies are characterized by extensive genetic and phenotypic heterogeneity. Primary diseases of the myocardium associated with mechanical and/or electrical dysfunction are included within this large group of diseases [1]. Various classification schemes have been proposed, with the classification according to functional and morphologic features remaining the most useful for clinical practice [2]. According to this scheme, the following major forms of cardiomyopathies have been defined: dilated cardiomyopathy (DCM), hypertrophic cardiomyopathy (HCM), restrictive cardiomyopathy (RCM), left ventricular noncompaction cardiomyopathy (LVNC), arrhythmogenic right ventricular cardiomyopathy (ARVC), and primary arrhythmias including long QT syndrome (LQTS), Brugada syndrome, and catecholaminergic polymorphic ventricular tachycardia (CPVT) [1,3,4]. As the involved pathogenetic mechanisms have been unveiled over the years, it has been attempted to link the different phenotypic forms of cardiomyopathies with the underlying genetic causes [5]. Thus, for example, genes encoding for sarcomere proteins are considered to play the primary role in the development of HCM, while mutations in genes that encode desmosomal proteins are found in most cases of ARVC.

Nevertheless, the definition of such genotype–phenotype correlations is far from simple due to the phenotypic overlap between the different forms of cardiomyopathy, as well as the overlap between several disease gene groups [2,6]. Further factors that contribute to the complexity of genetics of inherited cardiomyopathies are mutational heterogeneity, modifier genes, multiple variants or polygenic causes, genetic interplay with environmental influences, etc. [7]. These factors, along with the variable expressivity and incomplete penetrance shown by most of the cardiomyopathy phenotypes, make genetic testing and result interpretation particularly challenging.

Another limiting factor of current genetic testing in cardiomyopathies is that not all genetic causes have yet been discovered. This is reflected by the mutation detection rate of gene panels involving known genes with established association with cardiomyopathies, which is much less than 100%. The current diagnostic yield of such gene panels is around 10–40% for DCM and does not exceed 60% in the case of HCM or RCM [6]. Though secondary causes can account for a part of the remaining cases, the presence of positive family history in unsolved cases suggests that novel genetic causes of cardiomyopathies remain to be discovered [8].

As sequencing technologies have evolved over the years, extended genetic testing methods like whole exome sequencing (WES) or whole genome sequencing have been gaining ground in clinical diagnostics. These methods offer the possibility of analyzing more genes than the ones included in routine gene panels. However, there is an ongoing debate in the literature regarding the utility of whole exome/genome sequencing [9]. Among the expressed considerations are difficulties in the interpretation of numerous variants of uncertain significance, as well as secondary findings [10]. Furthermore, concerns have been raised about the wrong implications of some genes in the genetic etiology of cardiomyopathies, that may lead to false positive results in clinical diagnostic settings [11].

In this study, we examine the diagnostic benefit of extended genetic analysis beyond the major genes included in routine panels and address some of the above-mentioned challenges in cardiogenetics. For this purpose, we performed comprehensive genetic analyses of 61 consecutive patients diagnosed with a major form of cardiomyopathy or primary arrhythmia syndrome. Exome sequencing was performed, and data were analyzed by a stepwise approach starting from a disease-specific gene panel up to the analysis of all the exome data. We report the diagnostic yield after each step and describe the occurrence and distribution of relevant genetic variants, as well as the spectrum of affected genes. We discuss the limitations of current variant classification schemes and suggest new categories for uncertain variants. In the era of increasing availability of next generation sequencing (NGS) in clinical settings, it is important to explore both the potential and challenges of the complex field of cardiogenetics.

## 2. Materials and Methods

### 2.1. Clinical Evaluation

Unrelated patients were consecutively investigated in the “Center for Inherited Cardiovascular Diseases” at the University Hospital Würzburg between 2017 and 2019. The study protocol and procedures received positive votes from the ethics committee of the Medical Faculty of the University of Würzburg (vote #29/17), which was approved on 16 May 2017. All participants provided written informed consent prior to any study participation.

The purpose of presentation was the clinical evaluation of the index case, family history evaluation, and genetic diagnostics. The clinical evaluation included medical history, family history, 12-lead electrocardiogram (ECG), 24 h ambulatory Holter monitoring, transthoracic two-dimensional echocardiography, cardiac magnetic resonance imaging (CMR) in some cases, a cardiac biopsy, cardiac catheter examination, or additional electrophysiological investigations. Sixty-one patients with a primary diagnosis of HCM, DCM/LVNC, RCM, ARVC, LQTS, Brugada syndrome, CPVT, or who had survived sudden cardiac death without structural heart disease were included in the study. Classification and diagnosis were made according to the scientific statement of the American Heart Association [1]. For the sub-cohort of 36 DCM/LVNC patients, a secondary cause—in particular coronary artery disease, a primary/structural valvular defect, and an acute myocarditis—was excluded.

### 2.2. Exome Sequencing

For genetic testing, we performed exome DNA sequencing using different library preparation kits by Illumina (San Diego, CA, USA) and IDT (Integrated DNA Technologies, Coralville, IA, USA) according to the latest updates in library preparation proposed by Illumina.

The first 11 patients (#1, #5, #6, #7, #8, #23, #32, #33, #40, #49, and #50) recruited in the study were sequenced using the clinical Exome TruSight One Sequencing Panel (Illumina), which contains 4811 genes associated with a clinical phenotype. For the remaining 50 patients of the cohort, whole exome sequencing was performed using the following kits: 1: Nextera Rapid Capture Exome Kit (Illumina) with the commercial hybridization probes designed by Illumina; and 2: Nextera Library Prep for Enrichment (Illumina) or Nextera Flex for Enrichment (Illumina) with hybridization probes designed by IDT (xGen-Exome-Research-Panel v1.0 by IDT).

The pooled libraries were sequenced paired-end (2 × 150 base pairs) on a NextSeq^®^ 500 sequencing system (Illumina, San Diego, CA, USA). A minimum read depth of 10x across the target regions was achieved in 97.9% with the clinical Exome TruSight One Sequencing Panel, 96.5% with the Illumina Exome probes, and 99.3% with the IDT xGen-Exome-Research-Panel v1.0 probes.

### 2.3. Bioinformatics and Workflow of Genetic Data Analysis

Data analysis was performed by GensearchNGS (PhenoSystems, Wallonia, Belgium). Genetic variants were called for their minor allele frequency (MAF), exon distance (±20 bp into intron), and quality (variant present in > 10% of the NGS reads, coverage > 5×). Called variants were categorized as splice site, missense, nonsense, or small deletions/insertions. As the study focused on rare Mendelian variants with dominant inheritance, we used the MAF cutoff of <0.001 proposed in the literature [12]. The pathogenicity predictions were made using Alamut (Interactive Biosoftware, Rouen, France), which combines different prediction tools, information from mutation/polymorphism databases, and the Human Gene Mutation Database (HGMD^®^), which collects literature information on all known (published) gene variants responsible for human inherited diseases. The Genome Aggregation Database (gnomAD v2.1) was used as genetic reference database for unaffected individuals (http://gnomad.broadinstitute.org/) [13].

The variants were classified according to the American College of Medical Genetics and Genomics (ACMG) Standards and Guidelines [14]. According to this classification scheme, variants in genes that cause Mendelian disorders are categorized as “pathogenic,” “likely pathogenic,” “uncertain significance,” “likely benign,” or “benign” on the basis of various aspects of variant evidence (e.g., population data, computational data, functional data, and segregation data). VUS (variants of uncertain significance) in this study were subclassified further in “VUS favor pathogenic,” those that remained inconclusive (VUS), and “VUS favor benign” (Supplementary Figure S1). “VUS favor pathogenic” were classified as those variants that could not be confirmed as likely pathogenic according to the ACMG criteria but whose characteristics indicated a pathogenic relevance (e.g., absent from controls or very low frequency, affecting genes with well-known association with the patient phenotype, and/or affecting mutational hotspots). “VUS favor benign” were classified as those variants whose characteristics indicated that they are more likely to be benign (relatively high MAF, affected gene not fitting the clinical phenotype, and weakly conserved amino acid for missense variants).

**Figure 1:**
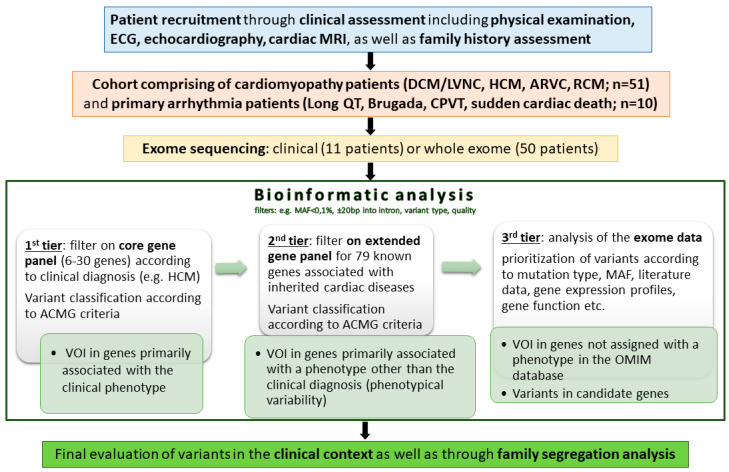
Workflow diagram. (DCM: dilated cardiomyopathy; HCM: hypertrophic cardiomyopathy; RCM: restrictive cardiomyopathy; LVNC: left ventricular noncompaction cardiomyopathy; ARVC: arrhythmogenic right ventricular cardiomyopathy; and CPVT: catecholaminergic polymorphic ventricular tachycardia).

The analysis of sequence data followed a three-step approach, as illustrated in the workflow diagram (Figure 1): In the first-tier analysis, variants were filtered for a gene panel according to the proposed clinical diagnosis (e.g., HCM; Supplementary Table S1). The gene selection for the panels was based on the recommendations of the American College of Medical Genetics and Genomics [6], the European Heart Rhythm Association [15], and the OMIM (Online Mendelian Inheritance in Man^®^) phenotype database. In a second step, an extended set of 79 genes (Supplementary Table S2; defined in 2017) associated with hereditary heart diseases in the OMIM database was screened for variants of interest (VOIs). This second step was performed in 40 patients in whom a likely pathogenic or pathogenic variant could not be detected in the first tier. “Variants of interest/VOIs” were considered pathogenic and likely pathogenic variants, as well as “VUS favor pathogenic.” In 32 patients in whom no VOIs could be detected in the second step, an analysis of the exome data followed. Variants in all genes enriched through exome sequencing were considered in the third tier. The prioritization of the single nucleotide variants (SNVs) in this third step was mainly based on the mutation type, MAF, conservation data for missense variants, literature data, and the function and expression profiles of the affected genes (http://www.proteinatlas.org [16]).

## 3. Results

### 3.1. Patient Characteristics

**Figure 2:**
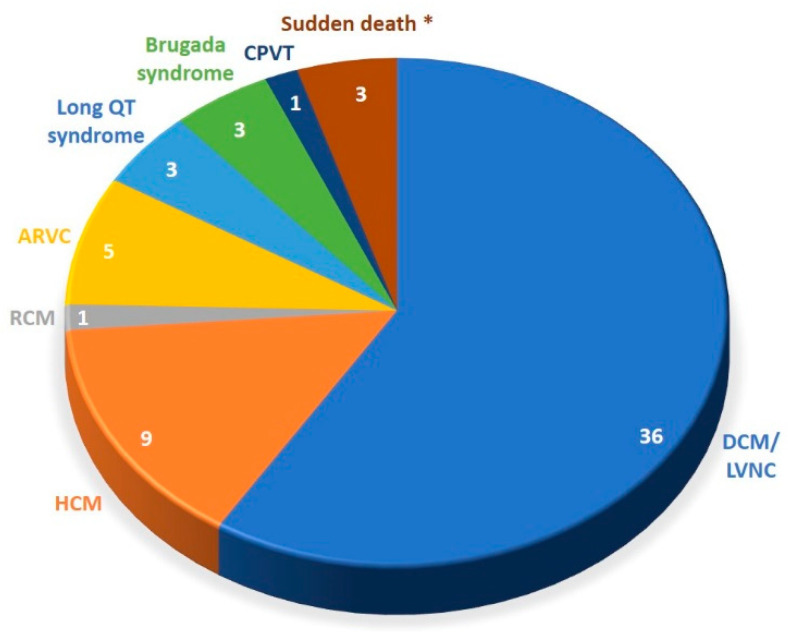
Clinical diagnoses of investigated patient cohort. * survived and non-survived.

The overall cohort consisted of 61 unrelated patients who were seen in a specialized clinic for inherited cardiac diseases for the purpose of clinical and family evaluation and genetic diagnostics. Fifty-one patients were diagnosed with a primary cardiomyopathy, with the majority of 36 patients with DCM or LVNC. Ten patients had a diagnosis of a primary arrhythmia syndrome, including three cases of survived sudden cardiac death without structural heart disease (Figure 2). The entire cohort had a mean age at first diagnosis of 37 ± 16 years, with 64% being male, and about half (48%) had an obvious family history of cardiomyopathy or arrhythmia in first- or second-degree relatives (Table 1).

The largest sub-cohort of 36 cases consisted of patients with DCM/LVNC. Here, the mean age of diagnosis was 37 ± 16 years, with 72% being males and 20 (56%) cases with a positive family history. About half of the cohort (47%) presented with chronic heart failure, while acute (decompensated) heart failure was the primary manifestation in about 44% of this cohort (Table 1). Echocardiographic measurements revealed a mean left ventricular ejection fraction (LVEF, Simpson biplane) of 32 ± 12% and a mean left ventricular end-diastolic diameter (LVEDD) of 65 ± 11 mm, thus indicating that most patients presented at an advanced stage of the disease.

### 3.2. Detection Rates of Variants of Interest and Spectrum of Incolved Genes

**Figure 3:**
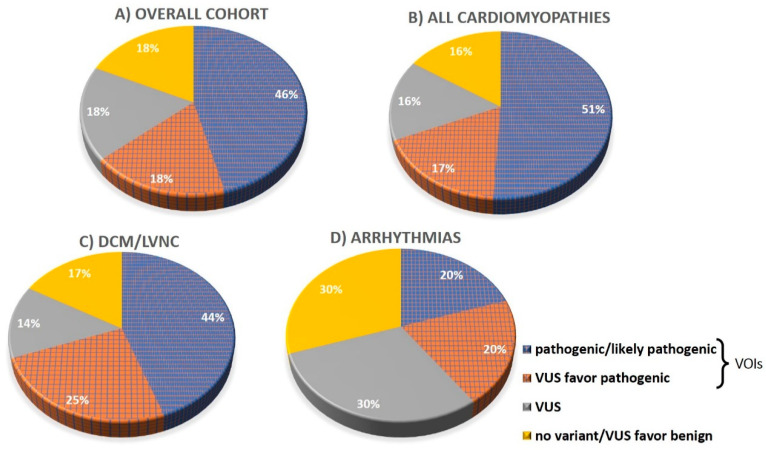
Detection rate of genetic variants in patient cohorts. (**A**) Overall cohort, (**B**) all cardiomyopathies, (**C**) DCM/LVNC, and (**D**) primary arrhythmias.

Overall, in 39 (64%) out of 61 patients of the entire cohort, a VOI could be detected including a pathogenic or likely pathogenic variant in 28 (46%) cases and a “VUS favor pathogenic” in 11 cases (Figure 3A). Twenty-three genetic variants appeared to be novel and were not previously described in the literature or in mutation databases (Table 2). Novel variants were submitted in the ClinVar database.

In the cohort of cardiomyopathy patients (DCM/LVNC, HCM, ARVC, LVNC, and RCM), the detection rate of VOIs was 68% (Figure 3B). The largest sub-cohort consisted of 36 DCM/LVNC patients. In this sub-cohort, a likely pathogenic/pathogenic variant was found in 16 (44%) of the patients, while “VUS favor pathogenic” could be detected in additional nine patients (25%), thus indicating an overall rate of VOIs of 25 (69%) (Figure 3C).

The highest detection rate was observed in the subgroup of patients with HCM, where a VOI was detected in seven out of nine cases. The lowest detection rate of VOIs (about 40%) was found in the primary arrhythmia patients (Figure 3D).

Regarding the involved genes, most detected variants in the DCM subgroup were truncating variants in the *TTN* gene. In accordance with previous reports [17], these truncating variants showed clustering in the A-band region of TTN and affected all major transcripts. One proband with a heterozygous truncating *TTN* variant had a clinical diagnosis of LVNC; an association of *TTN* variants with LVNC, although not established, was recently reported [18]. Genetic variants in further genes with established association for DCM/LVNC accounted for the rest of the cases: variants in genes coding for sarcomere proteins (e.g., *MYH7, MYBPC3,* and *TNNT2*), nuclear envelope proteins (e.g., *LMNA*), or the cytoskeleton (*FLNC*) (Supplementary Figure S2). Causative variants in genes encoding for desmosomal proteins (*DSP* and *DSG2*) that are primarily associated with ARVC were detected in two probands with a primary diagnosis of DCM, thus supporting the already reported association of those genes with DCM [19].

Most of the HCM-associated variants were identified in genes encoding for sarcomere proteins (*MYH7, MYBPC3,* and *TNNT2*), which is consistent with the established view that HCM is mainly a disease of the sarcomere (Supplementary Figure S2) [2].

The following interesting genotype–phenotype correlations were detected in the HCM group: One male proband with early onset HCM carried a hemizygous *FHL1* variant (patient #25). He had mildly elevated serum creatine kinase levels (~400 U/L) but no obvious skeletal muscle involvement. Isolated hypertrophic cardiomyopathy due to pathogenic *FHL1* mutations has been reported [20]. Further segregation studies revealed that his 50-year-old mother and 81-year-old grandmother were heterozygous carriers of the *FHL1* variant, both with mild signs of cardiac hypertrophy and diastolic dysfunction. In another patient with HCM (#23), a pathogenic missense variant in *MYH7* was detected in compound heterozygous state with a truncating splice variant in the same gene. A subsequent segregation analysis showed that the truncating variant was not associated with a clinical phenotype in the heterozygous state but led to a severe cardiomyopathy phenotype with non-compaction features in the compound heterozygous state [21].

Multiple variants could be also detected in five additional patients (pat. #3, #7, #22, #28, and #30; Table 2). Unlike patient #23, these multiple variants were found in different genes than the one harboring the primary causal variant. 

### 3.3. Diagnostic Yield of Core Gene Panel, Extended Gene Panel and Exome Analysis.

The highest diagnostic yield of VOIs was achieved in the core gene panel that fitted the clinical diagnosis (46%; 28/61). The panels contained all genes that were linked with the respective clinical phenotype in the OMIM database (Supplementary Table S1). In those cases, where no VOI was detected in the core gene panel, the analysis of an extended gene panel (Supplementary Table S2; defined in 2017) led to the detection of VOIs in an additional 13% of cases (8/61). This additional yield concerned cases with relevant variants in genes not primarily associated with clinical diagnosis, thus unveiling interesting genotype–phenotype correlations. For example, a likely pathogenic variant in *DSG2* was found in a proband with a clinical diagnosis of HCM (patient #27), suggesting a possible association with this phenotype.

A further analysis of the WES data led to the identification of VOIs in three cases, thus providing an additional diagnostic yield of about 5% (3/61). These cases involved variants in genes not well or only recently described in the literature (*TRIM63, MIB1,* and *MYLK3*) that were not included in the routine panel diagnostics at the time of the initial genetic analysis or were related to unusual inheritance patterns and phenotypes (Table 2 and Supplementary Table S3). In particular, an analysis of the whole exome data revealed a homozygous truncating variant in *TRIM63* (c.739C>T, p.Gln247 *) in an 18-year-old index patient of a consanguineous family (patient #45; Supplementary Figure S3). He was diagnosed with HCM (LVWT 27/24 mm) at the age of 12 years and got a defibrillator implanted after a syncopal episode. His sibling, who was also homozygous for the mutation, presented with a mild DCM phenotype at birth and also exhibited muscle pain and elevated serum creatine kinase levels. The index patient did not show an overt skeletal muscle phenotype but also had mildly elevated serum creatine kinase levels. Both heterozygous parents were healthy and did not show any signs of cardiomyopathy in echocardiographic studies. The detected *TRIM63* variant has been described in two literature reports in association with HCM and skeletal myopathy [22,23], but no OMIM phenotype had been assigned to the gene at the time of the genetic testing.

In another female patient (#44) with DCM and an early disease onset at the age of two years, a truncating *MIB1* variant (c.1111C>T, p.Arg371 *) could be detected. Her current age is 29 years, and she does not complain about heart failure symptoms under standard therapy. Her cardiac function with an LVEF of 42% has been stable for many years. Pathogenic *MIB1* mutations have been described as rare causes of LVNC [24], but they were not part of most routine gene panels at the time of testing.

Another 50-year-old patient with DCM (patient #15) carried a novel variant affecting a highly conserved amino acid residue in *MYLK3* (c.2042C>T, p.Pro681Leu). Loss-of-function variants in *MYLK3*, which codes for myosin light chain kinase 3, have just recently been described in association with DCM [25]. The same patient had an additional VUS in the *FLNC* gene (Table 2). However, we regard the *MYLK3* variant as more likely to be primarily causal because it affects a highly conserved amino acid residue at the catalytic domain of the encoded protein.

### 3.4. Novel Variants in Candidate Genes

Next, in unsolved cases, we prioritized variants of the exome data according to criteria illustrated in the workflow diagram (Figure 1). This allowed for the detection of variants in not well-studied genes whose expression and function profiles suggested a possible etiological relationship (Supplementary Table S3). The strongest candidate was a novel frameshift variant (c.222dup, p.Glu75Argfs * 16) in *STK38* encoding the serine-threonine kinase 38 in a 30-year-old patient presenting with acute heart failure, a severely dilated left ventricle (LVEDD: 75 mm), and a severely reduced left ventricular ejection fraction (LVEF: 19%). His family history revealed that his mother had a sudden cardiac arrest at the age of 59 years, but no autopsy was done. His maternal grandfather died at the age of about 40 years of unknown cause.

Recently, functional studies have shown that *STK38* interacts with *RBM24* (RNA binding motif protein 24), an RNA-binding protein with an important role in sarcomere assembly and heart contractility [26]. Furthermore, the knockdown of *STK38* led to the reduction of sarcomere proteins and the disarrangement of sarcomere [27]. *STK38* loss-of-function variants are infrequent and observed only in heterozygous form in population databases (<0.01%, https://gnomad.broadinstitute.org/). The gene shows a haploinsufficiency score of 2.67% (http://decipher.sanger.ac.uk) [28], suggesting an intolerance of loss-of-function variation.

## 4. Discussion

The identification of genetic causes of inherited cardiac diseases along with the improvement of DNA sequencing technologies have drastically changed cardiogenetics in the last years. Thus, genetic diagnostics have entered clinical routine and allow for the extensive testing of cardiomyopathy and arrhythmia patients. In this study, we performed a comprehensive genetic analysis of sequencing data starting from a targeted gene panel up to whole exome data in a stepwise approach. We reported on the additional diagnostic yield of an extended genetic analysis and propose novel candidate genes, but we also address some of the challenges encountered through this extensive data analysis approach and discuss the limitations of the ACMG variant classification criteria.

One of the challenges of extensive NGS diagnostics in inherited cardiac diseases is the classification of the numerous detected genetic variants and the interpretation of genetic findings in the clinical context. For this purpose, we used the ACMG criteria [14] in the first place. However, this classification scheme has some limitations, also reported in the literature [29], requiring revision and adaptation to the special features of different disease groups and genes. We observed in our study that three ACMG criteria in particular, i.e., PP1, PP5 (criteria with supporting evidence of pathogenicity), and PM2 (criterium with moderate evidence of pathogenicity), are sometimes difficult to apply. For example, a novel variant had not often been reported in mutation databases such as ClinVar or HGMD or in the literature before (PP5 criterium) or no family members were available for segregation analysis (PP1 criterium). Another frequently missing criterium was that, although very rare, variants were not completely absent from population databases, as demanded for the PM2 ACMG criterium [14]. In our view, this is a strong limitation, as pathogenic variants associated with diseases that show variable penetrance, like cardiomyopathies, may appear at a low frequency in control databases (i.e., <0.01%).

To overcome these limitations, we extended our classification of VUS by “VUS favor pathogenic” for variants missing one of these ACMG criteria (PP1, PP5, or PM2) (Supplementary Figure S1). A reclassification as “likely pathogenic” in some cases was possible after segregation studies or released literature/database reports. For example, a detected *FLNC* variant in patient #31 with RCM, could be reclassified as likely pathogenic, because the affected daughter was shown to be a carrier of the detected variant. Another example was a novel variant in the X-chromosomal *FHL1* gene found in a patient with HCM that was classified as “VUS favor pathogenic,” because segregation was initially missing and the variant was not described in the literature (missing PP5). This variant could be reclassified after confirmatory segregation analysis in likely pathogenic. Based on these examples, it is important to emphasize the significance of literature/database reports as well as segregation analysis for the interpretation of sequence variants. Overall, 7 out of the 11 VOIs missed a literature/database report at the time of the study in order to be classified as likely pathogenic (Table 2 and Supplementary Table S3). Four variants could not be classified as likely pathogenic because they were not completely absent from population databases, although they were only reported at a very low frequency (<0.01%). For example, a *TNNT2* variant in patient #11 (Table 2) could be regarded as likely pathogenic in our view, although it was not completely absent from population databases (MAF of ~0.001% in gnomAD).

In order to limit the number of reported variants to the most relevant ones, we also subclassified VUS with characteristics implying no clinical relevance (e.g., relatively high MAF, affected genes not fitting to the phenotype, and/or lacking sufficient evidence for a pathogenic role) as “VUS favor benign.” These variants are not reported in the manuscript.

In contrast to previous studies, the variant detection rate in the whole cohort, as well as in the DCM/LVNC sub-cohort, was higher [6,30], taking into account both pathogenic and likely pathogenic variants alone and even more pronounced when adding the subclass “VUS favor pathogenic”—together grouped as VOIs (Figure 3 and Table 1). This higher detection rate reflected the benefit of an extensive analysis approach that included not only the core genes for the respective clinical phenotype but also genes associated with all known cardiomyopathy or arrhythmia phenotypes. This allowed for the detection of variants of interest in genes other than the ones commonly associated with the clinical phenotype. In addition, we extended variant screening of the exome data to genes without a p-OMIM numbers at the time of testing based on the latest literature data and mutation databases (e.g., HGMD). Finally, a dedicated specialist for cardiogenetic conditions reviewed the variants in detail at the end of the process.

Another factor explaining the increased detection rates could be the fact that the examined patients were recruited in a specialized center for heart failure and cardiogenetics, although the recruitment criteria for genetic testing only excluded patients with known secondary causes of DCM such as ischemic, valvular, hypertensive, and acute inflammatory cardiomyopathy. We did not select for known family history of cardiomyopathy arrhythmia or sudden death. Interestingly, family history also did not show a statistically significant correlation with the variant detection rate (Fisher’s exact test: *p* = 0.857), indicating that family history alone may not be an indicator for genetic etiology.

In accordance with previous studies [9], the majority of VOIs were detected in disease-specific core gene panels (28/39 VOIs; 71%). However, 21% of VOIs (8/39 VOIs) were detected only after the extension of the analysis to genes not directly associated with the patient phenotype. This mainly concerned patients with a diagnosis of DCM who harbored a VOI in a gene coding for a desmosomal or a Z-disc (Filamin C, FLNC) protein, which demonstrated that overlapping phenotypes are a common issue in those patients. The benefit of expanded testing for DCM, which is characterized by extensive genetic heterogeneity, is in accordance with previous studies [30]. In our cohort though, a patient with a diagnosis of HCM carried a likely pathogenic variant in the desmosomal gene *DSG2*, an association that has thus far not been reported in the literature. It is important to emphasize here that the diagnostic benefit of the second step of our analysis mainly concerned genes with established pathogenic relevance that are primarily associated with a different clinical cardiac phenotype than the original clinical diagnosis. Though some studies have questioned the benefit of broad genetic diagnostics in inherited heart diseases [9] and have suggested limiting genetic testing to the core genes associated with a given phenotype [11], the extensive phenotypic variability of inherited cardiac diseases supports the requirement of at least an extended gene panel in routine diagnostics.

The weakness of targeted gene panel analysis was demonstrated in our study by the cases with the *TRIM63* and *MYLK3* variants. These genes had not been assigned a p-OMIM number at the time of analysis, and causal variants probably would have been missed if the analysis was confined to a gene panel. As genetic causes of cardiomyopathies continue to be discovered, it is important that panel diagnostics is not confined to genes linked with a phenotype in the OMIM database but also includes recently discovered genes, including the latest literature reports and current mutation databases (e.g., HGMD).

The need for extended genetic testing is also supported by the fact that multiple variants were detected in six patients, and these possibly modified the primary clinical phenotype. For example, in patient #28 with a primary clinical diagnosis of DCM, a pathogenic truncating variant in *TTN* was accompanied by a novel missense variant in *RYR2*. Interestingly, the patient showed an increased occurrence of ventricular arrhythmias. This exemplified the possibility that multiple variants could act as clinically relevant modifiers that are only detectable through expanded genetic analysis.

Since the additional diagnostic yield achieved by the analysis of the whole exome data was not extremely high, the utility in routine diagnostics can be questioned. Though the costs for consumables of exome sequencing are relatively low, the bioinformatic analysis and interpretation of the numerous variants are still time-consuming processes that require a dedicated specialist in the field of cardiogenetics. For as long as variant databases and bioinformatic tools remain imperfect and some genetic causes remain unrevealed, WES may not be practically applicable in daily clinical routines. However, as the identification of an underlying genetic cause of an inherited cardiac disease has implications for the surveillance of the patient and at-risk relatives, extensive genetic testing should be aimed at and ideally combined with a research project. Moreover, an analysis of whole exome data in research settings offers the possibility to identify candidate genes and thus unveil new genetic causes. In our study, we addressed this issue by expanding the analysis in unsolved cases and prioritized variants according to tissue expression profiles, knockout mouse model databases, and literature research, which led to the identification of variants in candidate genes, i.e., *STK38*. *STK38* is considered a strong candidate gene because of the variant type, as well as the population and current experimental data. Of course, the disease association should be thoroughly examined on the basis of functional and segregation studies. However, the chance of identification of these candidate genes would have been missed if the analysis was limited to a gene panel. Furthermore, the availability of exome data offers the possibility of the future reevaluation of the findings using new literature data.

However, our study had several limitations. First, the patient cohort was relatively small and heterogenous, with a wide spectrum of inherited cardiomyopathies and arrhythmias that did not allow for solid statistical conclusions. Furthermore, systematic family assessment was not performed to evaluate the segregation of VUS in all suspected cases, as well as the relevance of multiple variants with phenotypic severity. Apart from that, in those patients where a VOI was detected in the first tier, the analysis did not proceed to the second and third tier and so the presence of some multiple variants could have been missed. Finally, functional studies to prove the disease association of the proposed candidate genes were not part of this study.

## 5. Conclusions

Though the diagnostic yield of targeted gene panels can be considered as acceptable in a clinical setting, we favor extended genetic testing that makes use of the lately more readily available WES with subsequent thorough and stepwise analysis of the data. Using this approach, many of the challenges of genetic diagnostics in cardiogenetics—such as multiple variants, genetic heterogeneity, and phenotypic overlap—can be addressed. Of course, the challenge concerning the classification of the numerous detected variants increases with growing number of analyzed genes, thus highlighting the need to revise current classification schemes. However, as new causal genes for inherited cardiomyopathies are being described and some of the causes still remain undiscovered, it is important to extend the genetic analysis beyond targeted gene panels that contain a limited number of genes with established pathogenic relevance. The implementation of whole exome sequencing offers the possibility to identify variants in candidate genes, as well as the provision of data for a future analysis in a research setting. In this study, we clearly demonstrated the benefits of this approach in a cohort of 61 patients by describing new genotype–phenotype correlations, variants in not well-studied genes that would have been missed by a gene panel approach, and variants in novel candidate genes.

## Figures and Tables

**Table 1:** Sum statistics and genetics of the patient cohort.

Medical History	All *n* = 61	All CMP ^1^ *n* = 51	Primary Arrhythmias *n* = 10	DCM/LVNC ^2^ *n* = 36
Mean age at presentation, years (±SD)	42 ± 15.3	42 ± 15.1	39 ± 15.5	42 ± 15.4
Mean age of first diagnosis, years (±SD)	37 ± 15.6	37 ± 15.6	35 ± 15.1	37 ± 15.7
Males, *n* (%)	39 (64)	36 (70)	3 (30)	26 (72)
Family history of CMP^1^ or arrhythmia, *n* (%)	29 (47.5)	25 (49)	4 (40)	20 (55.5)
Family history of sudden death, *n* (%)	19 (31)	16 (31)	3 (30)	15 (42)
Presentation with ventricular arrhythmia or sudden death, *n* (%)	16 (26)	11 (22)	5 (50)	3 (8)
Presentation chronic heart failure, *n* (%)	18(29.5)	18 (35)	0	17 (47)
Presentation acute heart failure, *n* (%)	18 (29.5)	18 (35)	0	16 (44)
**Detected Variant**				
Pathogenic/likely pathogenic, *n* (%)	28 (46)	26 (51)	2 (20)	16 (44)
VUS ^3^ favor pathogenic, *n* (%)	11 (18)	9 (17)	2 (20)	9 (25)
VUS, *n* (%)	11 (18)	8 (16)	3 (30)	5 (14)
No variant found, *n* (%)	11 (18)	8 (16)	3 (30)	6 (17)
Variant of interest (VOI), *n* (%)	39 (64)	35 (68)	4 (40)	25 (69)

^1^ CMP: cardiomyopathies; ^2^ DCM: dilated cardiomyopathy. LVNC: left ventricular noncompaction cardiomyopathy; ^3^ VUS: variants of uncertain significance.

**Table 2:** Detected variants in genes assigned with a p-OMIM (Online Mendelian Inheritance in Man®) number at the time of testing.

Pat. #	Diagnosis	Gene	REFSEQ Transcript	HGVSc	HGVSp	Consequence	gnomAD AF	Popmax Filtering AF	ACMG Class	ACMG Rules	Missing Criterium for Classification as Likely Pathogenic ^§^
1	ARVC	*HCN4*	NM_005477.2	c.2266G>A	p.(Ala756Thr)	missense variant	0.00004923	0.00004798	variant of unknown significance	PP3	
2	ARVC	*CAV3*	NM_033337.2	c.233C>T	p.(Thr78Met)	missense variant	0.002667	0.003500	variant of unknown significance	PP2 PP3 PP5 BP6	
3	ARVC	*PKP2*	NM_004572.3	c.1237C>T	p.(Arg413 *)	nonsense variant	0.00001415		Pathogenic	PVS1 PP5	
		*PKP2*	NM_004572.3	c.2636T>C	p.(Leu879Pro)	missense variant	0.00001591	0.000007010	variant of unknown significance	PM2 PP3	
		*DSP*	NM_004415.3	c.5383T>A	p.(Ser1795Thr)	missense variant	0.00001062		variant of unknown significance	PM1 PM2	
4	ARVC	*PKP2*	NM_004572.3	c.369G>A	p.(Trp123 *)	nonsense variant	0.000004025		Pathogenic	PVS1 PP5	
5	DCM	*TNNT2*	NM_001001430.2	c.621G>C	p.(Lys207Asn)	missense variant			variant of unknown significance *	PM1 PM2 PP3	literature/database report (PP5)
6	DCM	*TTN*	NM_001267550.2	c.71083G>T	p.(Glu23695 *)	nonsense variant			Pathogenic	PVS1 PM2 PP4	
7	DCM	*TTN*	NM_001267550.2	c.101996G>A	p.(Trp33999 *)	nonsense variant			Pathogenic	PVS1 PM2 PP4	
		*TNNT2*	NM_001001430.2	c.83C>T	p.(Ala28Val)	missense variant	0.0004420	0.0005631	variant of unknown significance	PP5 BP6	
8	DCM	*TTN*	NM_001267550.2	c.5383A>T	p.(Lys1795 *)	nonsense variant			Pathogenic	PVS1 PM2 PP4	
9	DCM	*FLNC*	NM_001458.4	c.2504dup	p.(Pro836Thrfs * 84)	frameshift variant			Pathogenic	PVS1 PM2 PP4	
10	DCM	*TTN*	NM_001267550.2	c.75546C>A	p.(Tyr25182 *)	nonsense variant			Pathogenic	PVS1 PM2 PP4	
11	DCM	*TNNT2*	NM_001001430.2	c.406G>A	p.(Glu136Lys)	missense variant	0.00001066	0.000002930	variant of unknown significance *	PM1 PP3 PP5 PP4	variant not absent from controls(PM2)
12	DCM	*RBM20*	NM_001134363.2	c.1901G>T	p.(Arg634Leu)	missense variant			Pathogenic	PS3 PM2 PM5 PP3 PP5	
13	DCM	*DSP*	NM_004415.3	c.7570_7573del	p.(Thr2524Alafs*36)	frameshift variant			Pathogenic	PVS1 PM2 PP4	
14	DCM	*VCL*	NM_014000.2	c.1382C>A	p.(Ala461Asp)	missense variant			variant of unknown significance	PM2	
15	DCM	*FLNC*	NM_001458.4	c.4192A>G	p.(Lys1398Glu)	missense variant			variant of unknown significance	PM1 PM2 PP3	
16	DCM	*TTN*	NM_001267550.2	c.79684C>T	p.(Arg26562*)	nonsense variant			Pathogenic	PVS1 PM2 PP4 PP5	
17	DCM	*SCN5A*	NM_198056.2	c.1538G>C	p.(Arg513Pro)	missense variant	0.000004331		variant of unknown significance	PM2 BP4	
18	DCM	*SCN5A*	NM_198056.2	c.2441G>A	p.(Arg814Gln)	missense variant	0.00002507	0.000007170	likely pathogenic	PM1 PM4 PP3 PP5	
19	DCM	*LMNA*	NM_170707.3	c.555_556del	p.(Asp185Glufs * 9)	frameshift variant			Pathogenic	PVS1 PM2 PP4	
20	DCM	*EMD*	NM_000117.2	c.153dup	p.(Ser52Glnfs * 9)	frameshift variant			Pathogenic	PVS1 PM2 PP4 PP5	
21	DCM	*RYR2*	NM_001035.2	c.2026G>A	p.(Glu676Lys)	missense variant			variant of unknown significance *	PM1 PM2 PP3	literature/database report (PP5)
22	DCM	*RBM20*	NM_001134363.2	c.686A>G	p.(Tyr229Cys)	missense variant			variant of unknown significance	PM2 PP3	
		*MYH7*	NM_000257.3	c.3866G>A	p.(Arg1289Gln)	missense variant	0.00001061	0.000002920	variant of unknown significance *	PM1 PP2 PP3	variant not absent from controls(PM2)
23	HCM	*MYH7*	NM_000257.3	c.1207C>T	p.(Arg403Trp)	missense variant			Pathogenic	PS3 PM1 PM2 PM5 PP1 PP3 PP4 PP5	
		*MYH7*	NM_000257.3	c.1000-1G>A	p.?	splice variant	0.000007073		likely pathogenic	PM2 PP3 PP4	
24	HCM	*TNNT2*	NM_001001430.2	c.281G>T	p.(Arg94Leu)	missense variant			likely pathogenic	PS3 PM2 PP3 PP5	
25	HCM	*FHL1*	NM_001449.4	c.501G>C	p.(Lys167Asn)	missense variant			likely pathogenic	PM1 PM2 PP1 PP3	
26	HCM	*MYBPC3*	NM_000256.3	c.1440_1441delinsC	p.(Glu480Aspfs * 8)	frameshift variant			likely pathogenic	PVS1 PM2	
27	HCM	*DSG2*	NM_001943.4	c.593A>G	p.(Tyr198Cys)	missense variant	0.00001781	0.00002233	likely pathogenic	PM1 PM2 PP3 PP5	
28	DCM/Arrhythmias	*TTN*	NM_001267550.2	c.81341dup	p.(Asn27115Glufs * 10	frameshift variant			Pathogenic	PVS1 PM2 PP4	
		*RYR2*	NM_001035.2	c.322G>A	p.(Gly108Ser)	missense variant	0.00002271	0.000004630	variant of unknown significance	PM1 PM2 PP3	
29	LVNC	*MYH7*	NM_000257.3	c.3286G>T	p.(Asp1096Tyr)	missense variant	0.0001414	0.00005238	variant of unknown significance *	PM1 PP2 PP3 PP5	variant not absent from controls (PM2)
30	LVNC	*TTN*	NM_001267550.2	c.59848C>T	p.(Arg19950*)	nonsense variant			Pathogenic	PVS1 PM2 PP4	
		*MYH7*	NM_000257.3	c.5735T>A	p.(Ile1912Asn)	missense variant			variant of unknown significance	PM2 PP2 PP3	
31	RCM	*FLNC*	NM_001458.4	c.6031G>A	p.(Gly2011Arg)	missense variant			Likely pathogenic	PM1 PM2 PP1 PP3	
32	Long-QT/Arrhythmias	*KCNH2*	NM_000238.3	c.944T>C	p.(Leu315Ser)	missense variant			variant of unknown significance *	PM2 PP2 PP3 PP4	literature/database report (PP5)
33	Long-QT	*KCNQ1*	NM_000218.2	c.785T>C	p.(Leu262Pro)	missense variant			Likely pathogenic	PM1 PM2 PM5 PP3 PP5	
34	Brugada syndrome	*SCN5A*	NM_198056.2	c.4747C>T	p.(Arg1583Cys)	missense variant	0.000008026	0.000002940	Likely pathogenic	PM1 PM5 PP3 PP5	
35	DCM	*FLNC*	NM_001458.4	c.3275_3278delinsAAGA	p.(Thr1092_Gly1093delinsLysAsp)	in-frame delins			variant of unknown significance *	PM2 PP2 PP3 PP4	literature/database report (PP5)
36	Long-QT	*KCNH2*	NM_000238.3	c.526C>T	p.(Arg176Trp)	missense variant	0.0003237	0.0004289	variant of unknown significance	PP5	
37	DCM	*TNNI3K*	NM_015978.2	c.500T>C	p.(Phe167Ser)	missense variant	0.000007084		variant of unknown significance	PM1	
38	DCM	*MYBPC3*	NM_000256.3	c.2381C>T	p.(Pro794Leu)	missense variant	0.0001365	0.00002245	variant of unknown significance *	PM1 PP3 PP5	variant not absent from controls (PM2)
39	DCM	*MYH7*	NM_000257.3	c.1565A>T	p.(Asp522Val)	missense variant			variant of unknown significance *	PM1 PM2 PP2	literature/database report (PP5)
40	Survived sudden cardiac death	*CACNB2*	NM_201590.2	c.165A>T	p.(Lys55Asn)	missense variant			variant of unknown significance *	PM1 PM2 PP3	literature/database report (PP5)
41	DCM	*DSG2*	NM_001943.4	c.2533del	p.(Ile845 *)	frameshift variant			Likely pathogenic	PVS1 PM2 PP5	
42	HCM	*MYBPC3*	NM_000256.3	c.2454G>A	p.(Trp818 *)	nonsense variant			Pathogenic	PVS1 PM2 PP5	
43	DCM	*TTN*	NM_001267550.2	c.68022del	p.(Glu22675Lysfs * 7)	frameshift variant			Pathogenic	PVS1 PM2 PP4	
44	DCM	*MIB1*	NM_020774.3	c.1111C>T	p.(Arg371 *)	nonsense variant	0.00008856	0.00006398	Likely pathogenic	PVS1 PP1 PP4	

*: VUS favor pathogenic, ^§^ pertaining to VUS favor pathogenic. REFSEQ: Reference Sequence database. HGVSc: coding DNA reference sequence, HGVSp: protein reference sequence. Nomenclature according to the recommendations of the Human Genome Variation Society (HGVS). AF: Allele Frequency. p.?: unknown consequence at protein level. ACMG criteria for pathogenicity (for details see [14]): PVS1 (very strong evidence of pathogenicity), PM1/PM2/PM4/PM5 (moderate evidence of pathogenicity), PP1-PP5 (supporting evidence of pathogenicity).

## Supplementary Materials

The following are available online at https://www.mdpi.com/2077-0383/9/7/2168/s1, Table S1: Disease-specific gene panels, Table S2: Extended gene panel for cardiomyopathies and primary arrhythmia syndromes, Table S3: Detected variants in genes without a p-OMIM number at the time of testing, Figure S1: Variant classification scheme proposed in this study. Figure S2: Variants of interests according to molecular function in different patient sub-cohorts, Figure S3: Pedigree of the consanguineous family with the *TRIM63* variant.
