# Supplementary material for: New Insights on Genetic Diagnostics in Cardiomyopathy and Arrhythmia Patients Gained by Stepwise Exome Data Analysis
Source: J Clin Med. 2020 Jul 9;9(7):2168. doi: 10.3390/jcm9072168 (PMC7408654; doi:10.3390/jcm9072168)
Supplement: Supplementary file 1 [file jcm-09-02168-s001.pdf]

Journal of
Clinical Medicine

Supplemental Material

Table S1. Disease specific gene panels.

DCM panel						
**Gene Symbol**	**Location**	**Gene Description**	**RefSeq**	**ENSEMBL Transcript ID**	**Phenotype (Phenotype MIM Number)**	**Allelic Disorder (Phenotype MIM Number)**
ABCC9	12p12.1	ATP binding cassette subfamily C member 9	NM_005691.3	ENST00000261201.8	Cardiomyopathy, dilated, 1O (608569)	
ACTC1	15q11-q14	Actin alpha cardiac muscle 1	NM_005159.4	ENST00000290378.5	Cardiomyopathy, dilated, 1R (613424)	Cardiomyopathy, hypertrophic, 11 (612098)/Left ventricular noncompaction 4 (613424)
ACTN2	1q42-q43	Actinin alpha 2	NM_001103.3	ENST00000366578.5	Cardiomyopathy, dilated, 1AA, with or without LVNC (612158)	Cardiomyopathy, hypertrophic, 23, with or without LVNC (612158)
BAG3	10q25.2-q26.2	BCL2 associated athanogene 3	NM_004281.3	ENST00000369085.7	Cardiomyopathy, dilated, 1HH (613881)	Myopathy, myofibrillar, 6 (612954)
CRYAB	11q22.3-q23.1	Crystallin alpha B	NM_001885.2	ENST00000616970.4	Cardiomyopathy, dilated, 1II (615184)	Myopathy, myofibrillar, 2 (608810)
DES	2q35	Desmin	NM_001927.3	ENST00000373960.3	Cardiomyopathy, dilated, 1I (604765)	Myopathy, myofibrillar, 1 (601419)
DMD	Xp21.2	Dystrophin	NM_004006.2	ENST00000357033.8	Cardiomyopathy, dilated, 3B (302045)	Duchenne muscular dystrophy (310200)
DSG2	18q12.1	Desmoglein 2	NM_001943.4	ENST00000261590.12	Cardiomyopathy, dilated, 1BB (612877)	Arrhythmogenic right ventricular dysplasia 10 (610193)
DSP	6p24	Desmoplakin	NM_004415.3	ENST00000379802.7	Dilated cardiomyopathy with woolly hair, keratoderma, and tooth agenesis (615821)	Arrhythmogenic right ventricular dysplasia 8 (607450)
FKTN	9q31-q33	Fukutin	NM_001079802.1	ENST00000602661.5	Cardiomyopathy, dilated, 1X (611615)	Muscular dystrophy-dystroglycanopathy (congenital with brain and eye anomalies), type A, 4 (253800)

LAMA4	6q21	Laminin subunit alpha 4	NM_002290.4	ENST00000522006.5	Cardiomyopathy, dilated, 1JJ (615235)	
LDB3	10q22.3-q23.2	LIM domain binding 3	NM_001080116.1 NM_007078.2	ENST00000372066.7 ENST00000361373.8	Cardiomyopathy, dilated, 1C, with or without LVNC (601493)	Cardiomyopathy, hypertrophic, 24 (601493) / Myopathy, myofibrillar, 4 (609452)
LMNA	1q21.2-q21.3	Lamin A/C	NM_170707.3	ENST00000368300.8	Cardiomyopathy, dilated, 1A (115200)	Emery-Dreifuss muscular dystrophy 2, autosomal dominant (181350)
MYBPC3	11p11.2	Myosin binding protein C, cardiac	NM_000256.3	ENST00000545968.5	Cardiomyopathy, dilated, 1MM (615396)	Cardiomyopathy, hypertrophic, 4 (115197) / Left ventricular noncompaction 10 (615396)
MYH6	14q12	Myosin heavy chain 6	NM_002471.3	ENST00000405093.7	Cardiomyopathy, dilated, 1EE (613252)	Cardiomyopathy, hypertrophic, 14 (613251)
MYH7	14q12	Myosin heavy chain 7	NM_000257.3	ENST00000355349.3	Cardiomyopathy, dilated, 1S (613426)	Cardiomyopathy, hypertrophic, 1 (192600)
MYPN	10q21.3	Myopalladin	NM_032578.3	ENST00000358913.9	Cardiomyopathy, dilated, 1KK (615248)	Cardiomyopathy, hypertrophic, 22 (615248)
NEXN	1p31.1	Nexilin F-actin binding protein	NM_144573.3	ENST00000334785.11	Cardiomyopathy, dilated, 1CC (613122)	Cardiomyopathy, hypertrophic, 20 (613876)
PLN	6q22.1	Phospholamban	NM_002667.4	ENST00000357525.5	Cardiomyopathy, dilated, 1P (609909)	Cardiomyopathy, hypertrophic, 18 (613874)
RAF1	3p25	Raf-1 proto-oncogene, serine/threonine kinase	NM_002880.3	ENST00000251849.8	Cardiomyopathy, dilated, 1NN (615916)	Noonan syndrome 5 (611553)
RBM20	10q25.2	RNA binding motif protein 20	NM_001134363.2	ENST00000369519.3	Cardiomyopathy, dilated, 1DD (613172)	
SCN5A	3p21	Sodium voltage-gated channel alpha subunit 5	NM_198056.2	ENST00000333535.8	Cardiomyopathy, dilated, 1E (601154)	Brugada syndrome 1 (601144) / Long QT syndrome-3 (603830)
SDHA	5p15	Succinate dehydrogenase	NM_004168.3	ENST00000264932.10	Cardiomyopathy, dilated, 1GG (613642)	Paragangliomas 5 (614165)

		complex flavoprotein subunit A				
SGCD	5q33-q34	Sarcoglycan delta	NM_000337.5	ENST00000337851.8	Cardiomyopathy, dilated, 1L (606685)	Muscular dystrophy, limb-girdle, autosomal recessive 6 (601287)
TNNC1	3p21.3-p14.3	Troponin C1, slow skeletal and cardiac type	NM_003280.2	ENST00000232975.7	Cardiomyopathy, dilated, 1Z (611879)	Cardiomyopathy, hypertrophic, 13 (613243)
TNNI3	19q13.4	Troponin I3, cardiac type	NM_000363.4	ENST00000344887.9	Cardiomyopathy, dilated, 1FF (613286)	Cardiomyopathy, hypertrophic, 7 (613690)
TNNT2	1q32	Troponin T2, cardiac type	NM_001001430.2 NM_000364.3	ENST00000367318.9 ENST00000236918.11	Cardiomyopathy, dilated, 1D (601494)	Cardiomyopathy, hypertrophic, 2 (115195)
TPM1	15q22.1	Tropomyosin 1	NM_001018005.1	ENST00000403994.7	Cardiomyopathy, dilated, 1Y (611878)	Cardiomyopathy, hypertrophic, 3 (115196)
TTN	2q31	Titin	NM_001267550.2	ENST00000589042.5	Cardiomyopathy, dilated, 1G (604145)	
VCL	10q22.1-q23	Vinculin	NM_014000.2	ENST00000211998.9	Cardiomyopathy, dilated, 1W (611407)	Cardiomyopathy, hypertrophic, 15 (613255)

**HCM panel**						
**Gene Symbol**	**Location**	**Gene Description**	**RefSeq**	**ENSEMBL Transcript ID**	**Phenotype (Phenotype MIM Number)**	**Allelic Disorder (Phenotype MIM Number)**
ACTC1	15q11-q14	Actin alpha cardiac muscle 1	NM_005159.4	ENST00000290378.5	Cardiomyopathy, hypertrophic, 11 (612098)	Cardiomyopathy, dilated, 1R (613424) / Left ventricular noncompaction 4 (613424)
ACTN2	1q42-q43	Actinin alpha 2	NM_001103.3	ENST00000366578.5	Cardiomyopathy, hypertrophic, 23, with or without LVNC (612158)	Cardiomyopathy, dilated, 1AA, with or without LVNC (612158)

CAV3	3p25	Caveolin 3	NM_033337.2	ENST00000343849.2	Cardiomyopathy, familial hypertrophic (192600)	Long QT syndrome 9 (611818) / Rippling muscle disease 2 (606072)
CSRP3	11p15.1	Cysteine and glycine rich protein 3	NM_003476.4	ENST00000533783.2	Cardiomyopathy, hypertrophic, 12 (612124)	
FHL1	Xq26	Four and a half LIM domains 1	NM_001449.4	ENST00000370690.7	Emery-Dreifuss muscular dystrophy 6, X-linked (300696)	
GLA	Xq22	Galactosidase alpha	NM_000169.2	ENST00000218516.3	Fabry disease (301500)	
JPH2	20q13.12	Junctophilin 2	NM_020433.4	ENST00000372980.3	Cardiomyopathy, hypertrophic, 17 (613873)	
LAMP2	Xq24	Lysosomal associated membrane protein 2	NM_002294.2<br>NM_013995.2	ENST00000200639.8<br>ENST00000371335.4	Danon disease (300257)	
LDB3	10q22.3-q23.2	LIM domain binding 3	NM_001080116.1<br>NM_007078.2	ENST00000372066.7<br>ENST00000361373.8	Cardiomyopathy, hypertrophic, 24 (601493)	Cardiomyopathy, dilated, 1C, with or without LVNC (601493) / Myopathy, myofibrillar, 4 (609452)
MYBPC3	11p11.2	Myosin binding protein C, cardiac	NM_000256.3	ENST00000545968.5	Cardiomyopathy, hypertrophic, 4 (115197)	Cardiomyopathy, dilated, 1MM (615396) / Left ventricular noncompaction 10 (615396)
MYH6	14q12	Myosin heavy chain 6	NM_002471.3	ENST00000405093.7	Cardiomyopathy, hypertrophic, 14 (613251)	Cardiomyopathy, dilated, 1EE (613252)
MYH7	14q12	Myosin heavy chain 7	NM_000257.3	ENST00000355349.3	Cardiomyopathy, hypertrophic, 1 (192600)	Cardiomyopathy, dilated, 1S (613426)
MYL2	12q23-q24.3	Myosin light chain 2	NM_000432.3	ENST00000228841.12	Cardiomyopathy, hypertrophic, 10 (608758)	

MYL3	3p21.3-p21.2	Myosin light chain 3	NM_000258.2	ENST00000292327.4	Cardiomyopathy, hypertrophic, 8 (608751)	
MYLK2	20q13.31	Myosin light chain kinase 2	NM_033118.3	ENST00000375985.4	Cardiomyopathy, hypertrophic, 1, digenic (192600)	
MYOZ2	4q26-q27	Myozenin 2	NM_016599.4	ENST00000307128.5	Cardiomyopathy, hypertrophic, 16 (613838)	
MYPN	10q21.3	Myopalladin	NM_032578.3	ENST00000358913.9	Cardiomyopathy, hypertrophic, 22 (615248)	Cardiomyopathy, dilated, 1KK (615248)
NEXN	1p31.1	Nexilin F-actin binding protein	NM_144573.3	ENST00000334785.11	Cardiomyopathy, hypertrophic, 20 (613876)	Cardiomyopathy, dilated, 1CC (613122)
PLN	6q22.1	Phospholamban	NM_002667.4	ENST00000357525.5	Cardiomyopathy, hypertrophic, 18 (613874)	Cardiomyopathy, dilated, 1P (609909)
PRKAG2	7q36.1	Protein kinase AMP-activated non-catalytic subunit gamma 2	NM_016203.3	ENST00000287878.8	Cardiomyopathy, hypertrophic 6 (600858)	
TCAP	17q12	Titin-cap	NM_003673.3	ENST00000309889.2	Cardiomyopathy, hypertrophic, 25 (607487)	Muscular dystrophy, limb-girdle, autosomal recessive 7 (601954)
TNNC1	3p21.3-p14.3	Troponin C1, slow skeletal and cardiac type	NM_003280.2	ENST00000232975.7	Cardiomyopathy, hypertrophic, 13 (613243)	Cardiomyopathy, dilated, 1Z (611879)
TNNI3	19q13.4	Troponin I3, cardiac type	NM_000363.4	ENST00000344887.9	Cardiomyopathy, hypertrophic, 7 (613690)	Cardiomyopathy, dilated, 1FF (613286)

TNNT2	1q32	Troponin T2, cardiac type	NM_001001430.2 NM_000364.3	ENST00000367318.9 ENST00000236918.11	Cardiomyopathy, hypertrophic, 2 (115195)	Cardiomyopathy, dilated, 1D (601494)
TPM1	15q22.1	Tropomyosin 1	NM_001018005.1	ENST00000403994.7	Cardiomyopathy, hypertrophic, 3 (115196)	Cardiomyopathy, dilated, 1Y (611878)
TTN	2q31	Titin	NM_001267550.2	ENST00000589042.5	Cardiomyopathy, familial hypertrophic, 9 (613765)	Cardiomyopathy, dilated, 1G (604145)
TTR	18q12.1	Transthyretin	NM_000371.3	ENST00000237014.7	Amyloidosis, hereditary, transthyretin-related (105210)	
VCL	10q22.1-q23	Vinculin	NM_014000.2	ENST00000211998.9	Cardiomyopathy, hypertrophic, 15 (613255)	Cardiomyopathy, dilated, 1W (611407)

RCM panel

Gene Symbol	Location	Gene Description	RefSeq	ENSEMBL Transcript ID	Phenotype (Phenotype MIM Number)	Allelic Disorder (Phenotype MIM Number)
DES	2q35	Desmin	NM_001927.3	ENST00000373960.3		Cardiomyopathy, dilated, 1I (604765)/Myopathy, myofibrillar, 1 (601419)
FLNC	7q32-q35	Ffilamin C	NM_001458.4	ENST00000325888.12	Cardiomyopathy, familial restrictive 5 (617047)	Myopathy, myofibrillar, 5 (609524)
MYH7	14q12	Myosin heavy chain 7	NM_000257.3	ENST00000355349.3		Cardiomyopathy, hypertrophic, 1 (192600) / Cardiomyopathy, dilated, 1S (613426)
MYPN	10q21.3	Myopalladin	NM_032578.3	ENST00000358913.9	Cardiomyopathy, familial restrictive, 4 (615248)	Cardiomyopathy, hypertrophic, 22 (615248) / Cardiomyopathy, dilated, 1KK (615248)

TNNI3	19q13.4	Troponin I3, cardiac type	NM_000363.4	ENST00000344887.9	Cardiomyopathy, familial restrictive, 1 (115210)	Cardiomyopathy, hypertrophic, 7 (613690) / Cardiomyopathy, dilated, 1FF (613286)
TNNT2	1q32	Troponin T2, cardiac type	NM_001001430.2 NM_000364.3	ENST00000367318.9 ENST00000236918.11	Cardiomyopathy, familial restrictive, 3 (612422)	Cardiomyopathy, dilated, 1D (601494) / Cardiomyopathy, hypertrophic, 2 (115195)
TPM1	15q22.1	Tropomyosin 1	NM_001018005.1	ENST00000403994.7		Cardiomyopathy, hypertrophic, 3 (115196) / Cardiomyopathy, dilated, 1Y (611878)

**LVNC panel**						
**Gene Symbol**	**Location**	**Gene Description**	**RefSeq**	**ENSEMBL Transcript ID**	**Phenotype (Phenotype MIM Number)**	**Allelic Disorder (Phenotype MIM Number)**
ACTC1	15q11-q14	Actin alpha cardiac muscle 1	NM_005159.4	ENST00000290378.5	Left ventricular noncompaction 4 (613424)	Cardiomyopathy, hypertrophic, 11 (612098) / Cardiomyopathy, dilated, 1R (613424)
DES	2q35	Desmin	NM_001927.3	ENST00000373960.3		Cardiomyopathy, dilated, 1I (604765) / Myopathy, myofibrillar, 1 (601419)
DTNA	18q12	Dystrobrevin alpha	NM_001390.4	ENST00000444659.5	Left ventricular noncompaction 1, with or without congenital heart defects (604169)	
LDB3	10q22.3-q23.2	LIM domain binding 3	NM_001080116.1 NM_007078.2	ENST00000372066.7 ENST00000361373.8	Left ventricular noncompaction 3 (601493)	Cardiomyopathy, dilated, 1C, with or without LVNC (601493) / Cardiomyopathy, hypertrophic, 24 (601493) / Myopathy, myofibrillar, 4 (609452)
LMNA	1q21.2-q21.3	Lamin A/C	NM_170707.3	ENST00000368300.8		Emery-Dreifuss muscular dystrophy 2, autosomal dominant (181350) /

						Cardiomyopathy, dilated, 1A (115200)
MYH7	14q12	Myosin heavy chain 7	NM_000257.3	ENST00000355349.3	Left ventricular noncompaction 5 (613426)	Cardiomyopathy, dilated, 1S (613426) / Cardiomyopathy, hypertrophic, 1 (192600)
MYBPC3	11p11.2	Myosin binding protein C, cardiac	NM_000256.3	ENST00000545968.5	Left ventricular noncompaction 10 (615396)	Cardiomyopathy, dilated, 1MM (615396) /Cardiomyopathy, hypertrophic, 4 (115197)
PRDM16	1p36.23-p33	PR/SET domain 16	NM_022114.3	ENST00000270722.9	Left ventricular noncompaction 8 (615373)	
TAZ	Xq28	Tafazzin	NM_000116.4	ENST00000601016.5	Barth syndrome (302060)	
TNNT2	1q32	Troponin T2, cardiac type	NM_001001430.2 NM_000364.3	ENST00000367318.9 ENST00000236918.11	Left ventricular noncompaction 6 (601494)	Cardiomyopathy, dilated, 1D (601494) / Cardiomyopathy, hypertrophic, 2 (115195)
TPM1	15q22.1	Tropomyosin 1	NM_001018005.1	ENST00000403994.7	Left ventricular noncompaction 9 (611878)	Cardiomyopathy, dilated, 1Y (611878) / Cardiomyopathy, hypertrophic, 3 (115196)

**ARVC panel**						
**Gene Symbol**	**Location**	**Gene Description**	**RefSeq**	**ENSEMBL Transcript ID**	**Phenotype (Phenotype MIM Number)**	**Allelic Disorder (Phenotype MIM Number)**
CTNNA3	10q22.2	Catenin alpha 3	NM_013266.3	ENST00000433211.6	Arrhythmogenic right ventricular dysplasia, familial, 13 (615616)	
DES	2q35	Desmin	NM_001927.3	ENST00000373960.3		Cardiomyopathy, dilated, 1I (604765) / Myopathy, myofibrillar, 1 (601419)
DSC2	18q12.1	Desmocollin 2	NM_024422.4	ENST00000280904.10	Arrhythmogenic right ventricular dysplasia 11 (610476)	

DSG2	18q12.1	Desmoglein 2	NM_001943.4	ENST00000261590.12	Arrhythmogenic right ventricular dysplasia 10 (610193)	Cardiomyopathy, dilated, 1BB (612877)
DSP	6p24	Desmoplakin	NM_004415.3	ENST00000379802.7	Arrhythmogenic right ventricular dysplasia 8 (607450)	
JUP	17q21	Junction plakoglobin	NM_002230.3	ENST00000393931.7	Arrhythmogenic right ventricular dysplasia 12 (611528)	
LMNA	1q21.2-q21.3	Lamin A/C	NM_170707.3	ENST00000368300.8		Emery-Dreifuss muscular dystrophy 2, autosomal dominant (181350) / Cardiomyopathy, dilated, 1A (115200)
PKP2	12p11	Plakophilin 2	NM_004572.3	ENST00000070846.10	Arrhythmogenic right ventricular dysplasia 9 (609040)	
PLN	6q22.1	Phospholamban	NM_002667.4	ENST00000357525.5		Cardiomyopathy, dilated, 1P (609909) / Cardiomyopathy, hypertrophic, 18 (613874)
RYR2	1q42.1-q43	Ryanodine receptor 2	NM_001035.2	ENST00000366574.6	Arrhythmogenic right ventricular dysplasia 2 (600996)	Ventricular tachycardia, catecholaminergic polymorphic, 1 (604772)
TGFB3	14q24	Transforming growth factor beta 3	NM_003239.4	ENST00000238682.7	Arrhythmogenic right ventricular dysplasia 1 (107970)	
TMEM43	3p25.1	Transmembrane protein 43	NM_024334.2	ENST00000306077.4	Arrhythmogenic right ventricular dysplasia 5 (604400)	
TTN	2q31	Titin	NM_001267550.2	ENST00000589042.5		Cardiomyopathy, dilated, 1G (604145) / Cardiomyopathy, familial hypertrophic, 9 (613765)

Long QT panel						
**Gene Symbol**	**Location**	**Gene Description**	**RefSeq**	**ENSEMBL Transcript ID**	**Phenotype (Phenotype MIM Number)**	**Allelic Disorder (Phenotype MIM Number)**
AKAP9	7q21-q22	A-kinase anchoring protein 9	NM_005751.4	ENST00000356239.7	?Long QT syndrome-11 (611820)	
ANK2	4q25-q27	Ankyrin 2	NM_001148.5	ENST00000357077.8	Long QT syndrome 4 (600919)	
CACNA1C	12p13.3	Calcium voltage-gated channel subunit alpha1 C	NM_000719.6	ENST00000399655.5	Long QT syndrome 8 (618447)	Brugada syndrome 3 (611875)
CALM1	14q24-q31	Calmodulin 1	NM_006888.4	ENST00000356978.8	Long QT syndrome 14 (616247)	
CAV3	3p25	Caveolin 3	NM_033337.2	ENST00000343849.2	Long QT syndrome 9 (611818)	
KCNE1	21q22.12	Potassium voltage-gated channel subfamily E regulatory subunit 1	NM_000219.5	ENST00000399286.2	Long QT syndrome 5 (613695)	Jervell and Lange-Nielsen syndrome 2 (612347)
KCNE2	21q22.12	Potassium voltage-gated channel subfamily E regulatory subunit 2	NM_172201.1	ENST00000290310.3	Long QT syndrome 6 (613693)	
KCNJ2	17q23.1-q24.2	Potassium voltage-gated channel subfamily J member 2	NM_000891.2	ENST00000243457.3	Andersen syndrome/ Long QT syndrome 7 (170390)	
KCNJ5	11q24	Potassium voltage-gated channel subfamily J member 5	NM_000890.4	ENST00000529694.5	Long QT syndrome 13 (613485)	
KCNH2	7q35-q36	Potassium voltage-gated channel subfamily H member 2	NM_000238.3	ENST00000262186.9	Long QT syndrome 2 (613688)	Short QT syndrome 1 (609620)
KCNQ1	11p15.5	Potassium voltage-gated channel subfamily Q member 1	NM_000218.2	ENST00000155840.10	Long QT syndrome 1 (192500)	Jervell and Lange-Nielsen syndrome (220400) / Short QT syndrome 2 (609621)

SCN4B	11q23.3	Sodium voltage-gated channel beta subunit 4	NM_174934.3	ENST00000324727.8	Long QT syndrome-10 (611819)	
SCN5A	3p21	Sodium voltage-gated channel alpha subunit 5	NM_198056.2	ENST00000333535.8	Long QT syndrome-3 (603830)	Brugada syndrome 1 (601144)
SNTA1	20q11.2	Syntrophin alpha 1	NM_003098.2	ENST00000217381.2	Long QT syndrome 12 (612955)	

**Brugada panel**						
**Gene Symbol**	**Location**	**Gene Description**	**RefSeq**	**ENSEMBL Transcript ID**	**Phenotype (Phenotype MIM Number)**	**Allelic Disorder (Phenotype MIM Number)**
CACNA1C	12p13.3	Calcium voltage-gated channel subunit alpha1 C	NM_000719.6	ENST00000399655.5	Brugada syndrome 3 (611875)	Long QT syndrome 8 (618447)
CACNB2	10p12	Calcium voltage-gated channel auxiliary subunit beta 2	NM_201590.2	ENST00000377329.9	Brugada syndrome 4 (611876)	
GPD1L	3p22.3	Glycerol-3-phosphate dehydrogenase 1 like	NM_015141.3	ENST00000282541.9	Brugada syndrome 2 (611777)	
HCN4	15q24-q25	Hyperpolarization activated cyclic nucleotide gated potassium channel 4	NM_005477.2	ENST00000261917.3	Brugada syndrome 8 (613123)	
KCNE3	11q13-q14	Potassium voltage-gated channel subfamily E regulatory subunit 3	NM_005472.4	ENST00000310128.8	?Brugada syndrome 6 (613119)	
KCND3	1p13.3	Potassium voltage-gated channel subfamily D member 3	NM_004980.4	ENST00000315987.6	Brugada syndrome 9 (616399)	
SCN1B	19q13.1	Sodium voltage-gated channel beta subunit 1	NM_001037.4	ENST00000638536.1	Brugada syndrome 5 (612838)	
SCN3B	11q23.3	Sodium voltage-gated channel beta subunit 3	NM_018400.3	ENST00000392770.6	Brugada syndrome 7 (613120)	

SCN5A	3p21	Sodium voltage-gated channel alpha subunit 5	NM_198056.2	ENST00000333535.8	Brugada syndrome 1 (601144)	Long QT syndrome-3 (603830)
TRPM4	19q13.33	Transient receptor potential cation channel subfamily M member 4	NM_017636.3	ENST00000252826.9		Progressive familial heart block, type IB (604559)

**CPVT panel**						
**Gene Symbol**	**Location**	**Gene Description**	**RefSeq**	**ENSEMBL Transcript ID**	**Phenotype (Phenotype MIM Number)**	**Allelic Disorder (Phenotype MIM Number)**
CALM1	14q24-q31	Calmodulin 1	NM_006888.4	ENST00000356978.8	Ventricular tachycardia, catecholaminergic polymorphic, 4 (614916)	Long QT syndrome 14 (616247)
CASQ2	1p13.3-p11	Calsequestrin 2	NM_001232.3	ENST00000261448.5	Ventricular tachycardia, catecholaminergic polymorphic, 2 (611938)	
GNAI2	3p21	G protein subunit alpha i2	NM_002070.3	ENST00000313601.10	Ventricular tachycardia, idiopathic (192605)	
KCNJ2	17q23.1-q24.2	Potassium voltage-gated channel subfamily J member 2	NM_000891.2		ENST00000243457.3	Andersen syndrome/ Long QT syndrome 7 (170390)
RYR2	1q42.1-q43	Ryanodine receptor 2	NM_001035.2	ENST00000366574.6	Ventricular tachycardia, catecholaminergic polymorphic, 1 (604772)	Arrhythmogenic right ventricular dysplasia 2 (600996)
TRDN	6q22.31	Triadin	NM_006073.3	ENST00000334268.8	Ventricular tachycardia, catecholaminergic polymorphic, 5, with or without muscle weakness (615441)	

Table S2. Extended gene panel for cardiomyopathies and primary arrhythmia syndromes.

**Gen**	**Location**	**Gene Description**	**RefSeq**	**Transkript-ID Ensemble**	**p-OMIM**	**Allelic Disorder**
ABCC9	12p12.1	ATP binding cassette subfamily C member 9	**NM_005691.3**	ENST00000261201.8<br>ENST00000261200.8	Cardiomyopathy, dilated, 1O (608569)	

ACTC1	15q11-q14	Actin alpha cardiac muscle 1	**NM_005159.4**	ENST00000290378.5	Cardiomyopathy, hypertrophic, 11 612098	Cardiomyopathy, hypertrophic, 11 (612098) / Left ventricular noncompaction 4 (613424)
ACTN2	1q42-q43	Actinin alpha 2	**NM_001103.3**	ENST00000366578.5	Cardiomyopathy, dilated, 1AA, with or without LVNC (612158)	Cardiomyopathy, hypertrophic, 23, with or without LVNC (612158)
AKAP9	7q21-q22	A-kinase anchoring protein 9	**NM_005751.4**	ENST00000356239.7	?Long QT syndrome-11 (611820)	
ANK2	4q25-q27	Ankyrin 2	**NM_001148.5**	ENST00000357077.8	Long QT syndrome 4 (600919)	
BAG3	10q25.2-q26.2	BCL2 associated athanogene 3	**NM_004281.3**	ENST00000369085.7	Cardiomyopathy, dilated, 1HH (613881)	Myopathy, myofibrillar, 6 (612954)
CACNA1C	12p13.3	Calcium voltage-gated channel subunit alpha1 C	**NM_000719.6**	ENST00000399655.5 ENST00000347598.8	Long QT syndrome 8 (618447)	
CACNB2	10p12	Calcium voltage-gated channel auxiliary subunit beta 2	**NM_201590.2**	ENST00000377329.9 ENST00000324631.11	Brugada syndrome 4 (611876)	
CALM1	14q24-q31	Calmodulin 1	**NM_006888.4**	ENST00000356978.8	Long QT syndrome 14 616247	
CAV3	3p25	Caveolin 3	**NM_033337.2**	ENST00000343849.2	Long QT syndrome 9 (611818)	Cardiomyopathy, familial hypertrophic (192600) / Rippling muscle disease 2 (606072)
CASQ2	1p13.3-p11	Calsequestrin 2	**NM_001232.3**	ENST00000261448.5	Ventricular tachycardia, catecholaminergic polymorphic, 2 (611938)	
CRYAB	11q22.3-q23.1	Crystallin alpha B	**NM_001885.2**	ENST00000616970.4	Cardiomyopathy, dilated, 1II (615184)	Myopathy, myofibrillar, 2 (608810)
CSRP3	11p15.1	Cysteine and glycine rich protein 3	**NM_003476.4**	ENST00000533783.2	Cardiomyopathy, hypertrophic, 12 (612124)	

CTNNA3	10q22.2	Catenin alpha 3	**NM_013266.3**	ENST00000433211.6	Arrhythmogenic right ventricular dysplasia, familial, 13 (615616)	
DES	2q35	Desmin	**NM_001927.3**	ENST00000373960.3	Cardiomyopathy, dilated, 1I 604765	
DMD	Xp21.2	Dystrophin	**NM_004006.2**	ENST00000357033.8	Cardiomyopathy, dilated, 3B (302045)	Duchenne muscular dystrophy (310200)
DSC2	18q12.1	Desmocollin 2	**NM_024422.4** NM_004949.3	ENST00000280904.10 ENST00000251081.6	Arrhythmogenic right ventricular dysplasia 11 (610476)	
DSG2	18q12.1	Desmoglein 2	**NM_001943.4**	ENST00000261590.12	Arrhythmogenic right ventricular dysplasia 10 (610193)	Cardiomyopathy, dilated, 1BB (612877)
DSP	6p24	Ddesmoplakin	**NM_004415.3**	ENST00000379802.7	Arrhythmogenic right ventricular dysplasia 8 (607450)	Dilated cardiomyopathy with woolly hair, keratoderma, and tooth agenesis (615821)
DTNA	18q12	Dystrobrevin alpha	**NM_001390.4**	ENST00000444659.5	Left ventricular noncompaction 1, with or without congenital heart defects (604169)	
EMD	Xq28	Eemerin	**NM_000117.2**	ENST00000369842.8	Emery-Dreifuss muscular dystrophy 1, X-linked (310300)	
FHL1	Xq26	Four and a half LIM domains 1	**NM_001449.4**	ENST00000370690.7 ENST00000394155.6	Emery-Dreifuss muscular dystrophy 6, X-linked (300696)	
FKTN	9q31-q33	Fukutin	**NM_001079802.1**	ENST00000602661.5	Cardiomyopathy, dilated, 1X (611615)	Muscular dystrophy-dystroglycanopathy (congenital with brain and eye anomalies), type A, 4 (253800)
FLNC	7q32-q35	filamin C	**NM_001458.4**	ENST00000325888.12	Cardiomyopathy, familial restrictive 5 (617047)	
GATAD1	7q21-q22	GATA zinc finger domain containing 1	**NM_021167.4**	ENST00000287957.3	?Cardiomyopathy, dilated, 2B (614672)	

GLA	Xq22	Galactosidase alpha	**NM_000169.2**	ENST00000218516.3	Fabry disease (301500)	
GNAI2	3p21	G protein subunit alpha i2	**NM_002070.3**	ENST00000313601.10	Ventricular tachycardia, idiopathic (192605)	
GPD1L	3p22.3	Glycerol-3-phosphate dehydrogenase 1 like	**NM_015141.3**	ENST00000282541.9	Brugada syndrome 2 (611777)	
HCN4	15q24-q25	Hyperpolarization activated cyclic nucleotide gated potassium channel 4	**NM_005477.2**	ENST00000261917.3	Brugada syndrome 8 (613123)	
JPH2	20q13.12	Junctophilin 2	**NM_020433.4**	ENST00000372980.3	Cardiomyopathy, hypertrophic, 17 (613873)	
JUP	17q21	Junction plakoglobin	**NM_002230.3**	ENST00000393931.7	Arrhythmogenic right ventricular dysplasia 12 611528	
KCND3	1p13.3	Potassium voltage-gated channel subfamily D member 3	**NM_004980.4**	ENST00000315987.6	Brugada syndrome 9 (616399)	
KCNE1	21q22.12	Potassium voltage-gated channel subfamily E regulatory subunit 1	**NM_000219.5**	ENST00000399286.2	Long QT syndrome 5 (613695)	
KCNE2	21q22.12	Potassium voltage-gated channel subfamily E regulatory subunit 2	**NM_172201.1**	ENST00000290310.3	Long QT syndrome 6 (613693)	
KCNE3	11q13-q14	Potassium voltage-gated channel subfamily E regulatory subunit 3	**NM_005472.4**	ENST00000310128.8	?Brugada syndrome 6 (613119)	
KCNH2	7q35-q36	Potassium voltage-gated channel subfamily H member 2	**NM_000238.3**	ENST00000262186.9	Long QT syndrome 2 (613688)	
KCNJ2	17q23.1-q24.2	Potassium voltage-gated channel subfamily J member 2	**NM_000891.2**	ENST00000243457.3	Short QT syndrome 3 609622	Andersen syndrome/ Long QT syndrome 7 (170390)

KCNJ5	11q24	Potassium voltage-gated channel subfamily J member 5	**NM_000890.4**	ENST00000529694.5	Long QT syndrome 13 613485	
KCNQ1	11p15.5	Potassium voltage-gated channel subfamily Q member 1	**NM_000218.2**	ENST00000155840.10	Long QT syndrome 1 (192500)	
LAMA4			**NM_002290.4**	ENST00000522006.5 ENST00000230538.11	Cardiomyopathy, dilated, 1JJ (615235)	
LAMP2	Xq24	Lysosomal associated membrane protein 2	**NM_002294.2**	ENST00000200639.8 ENST00000371335.4 ENST00000434600.6	Danon disease (300257)	
LDB3	10q22.3-q23.2	LIM domain binding 3	**NM_007078.2 NM_001080116.1**	ENST00000361373.8 ENST00000372066.7 ENST00000263066.10	Cardiomyopathy, dilated, 1C, with or without LVNC (601493)	
LMNA	1q21.2-q21.3	Lamin A/C	**NM_170707.3**	ENST00000368300.8	Cardiomyopathy, dilated, 1A (115200)	Emery-Dreifuss muscular dystrophy 2, autosomal dominant (181350)
MYBPC3	11p11.2	Myosin binding protein C, cardiac	**NM_000256.3**	ENST00000545968.5	Cardiomyopathy, hypertrophic, 4 (115197)	Cardiomyopathy, dilated, 1MM (615396) / Left ventricular noncompaction 10 (615396)
MYH6	14q12	Myosin heavy chain 6	**NM_002471.3**	ENST00000405093.7	Cardiomyopathy, dilated, 1EE 613252	
MYH7	14q12	Myosin heavy chain 7	**NM_000257.3**	ENST00000355349.3	Cardiomyopathy, hypertrophic, 1 (192600)	Cardiomyopathy, dilated, 1S (613426)
MYL2	12q23-q24.3	Myosin light chain 2	**NM_000432.3**	ENST00000228841.12	Cardiomyopathy, hypertrophic, 10 608758	
MYL3	3p21.3-p21.2	Myosin light chain 3	**NM_000258.2**	ENST00000292327.4	Cardiomyopathy, hypertrophic, 8 (608751)	
MYLK2	20q13.31	Myosin light chain kinase 2	**NM_033118.3**	ENST00000375985.4	Cardiomyopathy, hypertrophic, 1, digenic (192600)	

MYOZ2	4q26-q27	Myozenin 2	**NM_016599.4**	ENST00000307128.5	Cardiomyopathy, hypertrophic, 16 (613838)	
MYPN	10q21.3	Myopalladin	**NM_032578.3**	ENST00000358913.9	Cardiomyopathy, hypertrophic, 22 (615248)	Cardiomyopathy, dilated, 1KK (615248)
NEXN	1p31.1	Nexilin F-actin binding protein	**NM_144573.3**	ENST00000334785.11	Cardiomyopathy, dilated, 1CC (613122)	Cardiomyopathy, hypertrophic, 20 (613876)
PKP2	12p11	Plakophilin 2	**NM_004572.3**	ENST00000070846.10	Arrhythmogenic right ventricular dysplasia 9 609040	
PLN	6q22.1	Phospholamban	**NM_002667.4**	ENST00000357525.5	Cardiomyopathy, dilated, 1P 609909	Cardiomyopathy, hypertrophic, 18 (613874)
PRDM16	1p36.23-p33	PR/SET domain 16	**NM_022114.3**	ENST00000270722.9	Left ventricular noncompaction 8 (615373)	
PRKAG2	7q36.1	Protein kinase AMP-activated non-catalytic subunit gamma 2	**NM_016203.3**	ENST00000287878.8	Cardiomyopathy, hypertrophic 6 (600858)	
RAF1	3p25	Raf-1 proto-oncogene, serine/threonine kinase	**NM_002880.3**	ENST00000251849.8	Cardiomyopathy, dilated, 1NN 615916	
RBM20	10q25.2	RNA binding motif protein 20	**NM_001134363.2**	ENST00000369519.3	Cardiomyopathy, dilated, 1DD (613172)	
RYR2	1q42.1-q43	Ryanodine receptor 2	**NM_001035.2**	ENST00000366574.6	Ventricular tachycardia, catecholaminergic polymorphic, 1 604772	
SCN1B	19q13.1	Sodium voltage-gated channel beta subunit 1	**NM_001037.4**	ENST00000638536.1 ENST00000415950.4	Brugada syndrome 5 (612838)	
SCN3B	11q23.3	Sodium voltage-gated channel beta subunit 3	**NM_018400.3**	ENST00000392770.6	Brugada syndrome 7 (613120)	
SCN4B	11q23.3	Sodium voltage-gated channel beta subunit 4	**NM_174934.3**	ENST00000324727.8	Long QT syndrome-10 (611819)	
SCN5A	3p21	Sodium voltage-gated channel alpha subunit 5	**NM_198056.2**	ENST00000333535.8	Brugada syndrome 1 (601144) / Long QT syndrome-3 (603830)	Cardiomyopathy, dilated, 1E (601154)
SDHA	5p15	Succinate dehydrogenase complex flavoprotein subunit A	**NM_004168.3**	ENST00000264932.10	Cardiomyopathy, dilated, 1GG (613642)	Paragangliomas 5 (614165)

SGCD	5q33-q34		**NM_000337.5**	ENST00000337851.8	Cardiomyopathy, dilated, 1L (606685)	Muscular dystrophy, limb-girdle, autosomal recessive 6 (601287)
SNTA1	20q11.2	Syntrophin alpha 1	**NM_003098.2**	ENST00000217381.2	Long QT syndrome 12 (612955)	
TAZ	Xq28	Tafazzin	**NM_000116.4**	ENST00000601016.5	Barth syndrome (302060)	
TCAP	17q12	Titin-cap	**NM_003673.3**	ENST00000309889.2	Cardiomyopathy, hypertrophic, 25 (607487)	Muscular dystrophy, limb-girdle, autosomal recessive 7 (601954)
TGFB3	14q24	Transforming growth factor beta 3	**NM_003239.4**	ENST00000238682.7	Arrhythmogenic right ventricular dysplasia 1 107970	
TMEM43	3p25.1	Transmembrane protein 43	**NM_024334.2**	ENST00000306077.4	Arrhythmogenic right ventricular dysplasia 5 604400	
TNNC1	3p21.3-p14.3	Troponin C1, slow skeletal and cardiac type	**NM_003280.2**	ENST00000232975.7	Cardiomyopathy, dilated, 1Z (611879)	Cardiomyopathy, hypertrophic, 13 (613243)
TNNI3	19q13.4	Troponin I3, cardiac type	**NM_000363.4**	ENST00000344887.9	Cardiomyopathy, dilated, 1FF (613286)	Cardiomyopathy, hypertrophic, 7 (613690)
TNNT2	1q32	Troponin T2, cardiac type	**NM_001001430.2**	ENST00000367318.9<br>ENST00000236918.11	Cardiomyopathy, dilated, 1D (601494)	Cardiomyopathy, hypertrophic, 2 (115195)
TPM1	15q22.1	Tropomyosin 1	**NM_001018005.1**	ENST00000403994.7<br>ENST00000559556.5	Cardiomyopathy, hypertrophic, 3 (115196)	Cardiomyopathy, dilated, 1Y (611878)
TRDN	6q22.31	Triadin	**NM_006073.3**	ENST00000334268.8	Ventricular tachycardia, catecholaminergic polymorphic, 5, with or without muscle weakness (615441)	
TRPM4	19q13.33	Transient receptor potential cation channel subfamily M member 4	**NM_017636.3**	ENST00000252826.9	Progressive familial heart block, type IB (604559)	
TTN	2q31	Titin	**NM_001267550.2**	ENST00000589042.5<br>ENST00000342992.10	Cardiomyopathy, dilated, 1G (604145)	
TTR	18q12.1	Transthyretin	**NM_000371.3**	ENST00000237014.7	Amyloidosis, hereditary, transthyretin-related (105210)	

VCL	10q22.1-q23	Vinculin	**NM_014000.2**	ENST00000211998.9	Cardiomyopathy, dilated, 1W (611407)	Cardiomyopathy, hypertrophic, 15 (613255)

Table S3. Detected variants in genes without a p-OMIM number at the time of testing.

Pat. #	Diagnosis	Gene	REFSEQ	HGVSc	HGVSp	Consequence	gnomAD AF	Popmax Filtering AF	ACMG Class	ACMG Rules	Pathogenicity Clues
**Genes with evidence of pathogenic relevance**											
**15**	DCM	*MYLK3*	NM_182493.2	c.2042C>T	p.(Pro681Leu)	missense variant			variant of unknown significance*	PM1, PM2, PP4 (no literature/database report)	Identification of *MYLK3* mutations in familial dilated cardiomyopathy [1]
**45**	HCM	*TRIM63*	NM_032588.3	c.739C>T	p.(Gln247*)	nonsense variant	0.0006787	0.0003963	likely pathogenic	PVS1, PP4, PP5	[2,3]
Not well studied candidate genes											
**6**	HCM	*ARNTL*	NM_001297719.1	c.1588C>G	p.(Pro530Ala)	missense variant			n/a	n/a	Development of dilated cardiomyopathy in Bmal1-deficient mice [4]
**1**	ARVC	*OBSCN*	NM_052843.3	c.8243T>C	p.(Leu2748Pro)	missense variant	0.00002020	0.00001712	n/a	n/a	*OBSCN* Mutations Associated with Dilated Cardiomyopathy and Haploinsufficiency [5]
		MYOM1	NM_003803.3	c.776A>G	p.(Glu259Gly)	missense variant	0.00001835	0.00001147	n/a	n/a	Titin-associated Protein, Association with HCM [6]
**47**	DCM	*STK38*	NM_007271.3	c.222dup	p.(Glu75Argfs*16)	frameshift variant			n/a	n/a	Stk38 Modulates Rbm24 Protein Stability to

											Regulate Sarcomere Assembly in Cardiomyocytes [7]
**48**	Brugada syndrome	*SLMAP*	NM_007159.4	c.1663A>G	p.(Ser555Gly)	missense variant	0.000007960	0.00001898	n/a	n/a	Association with Brugada syndrome [8]

* : VUS favor pathogenic, n/a: not applicable.

Figure S1. Variant classification scheme proposed in this study. (VUS: variants of uncertain significance).

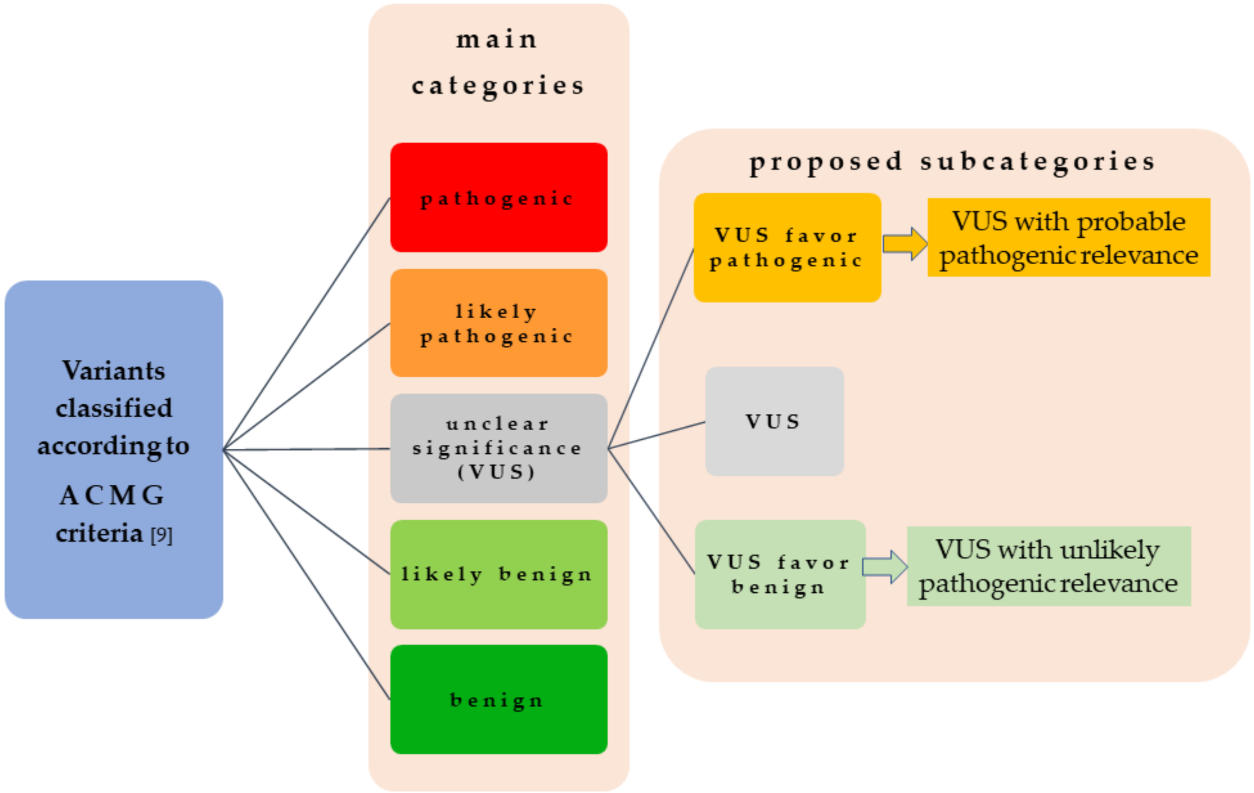

Figure S2. Variants of interests according to molecular function in different patient sub-cohorts.

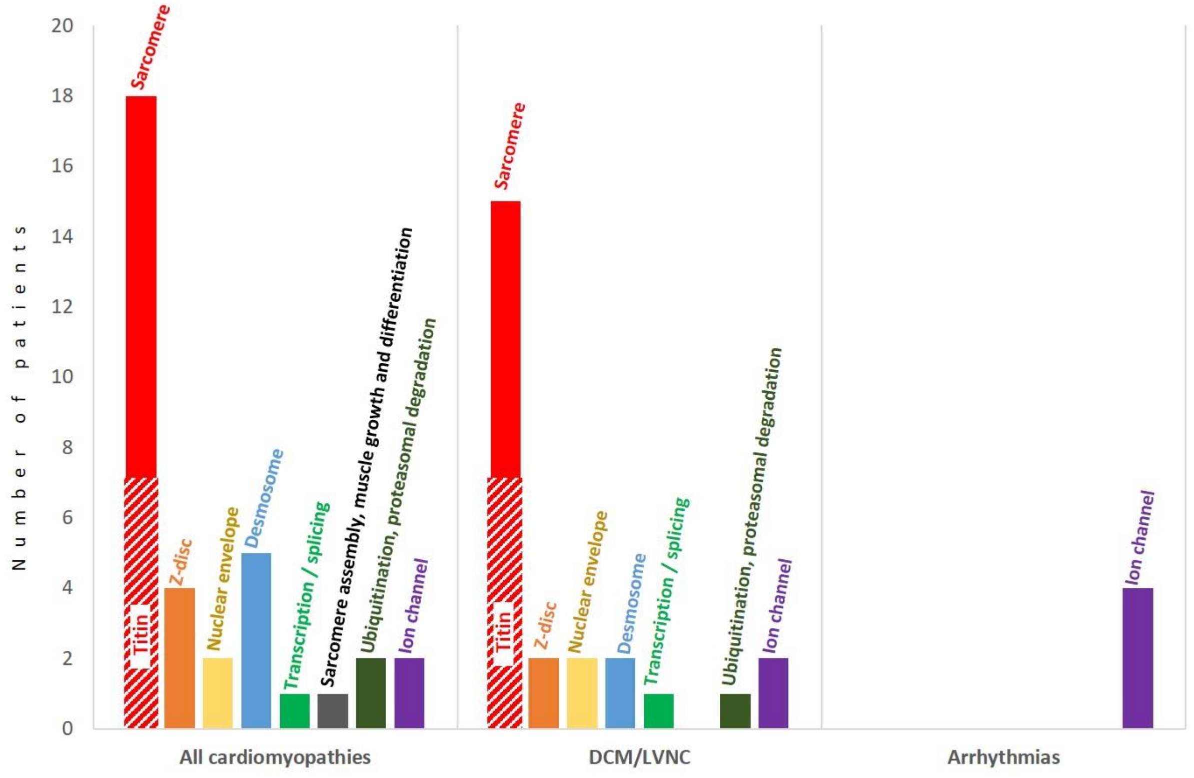

Figure S3. Pedigree of the consanguineous family (origin: Christian Arabs) with the *TRIM63* variant.

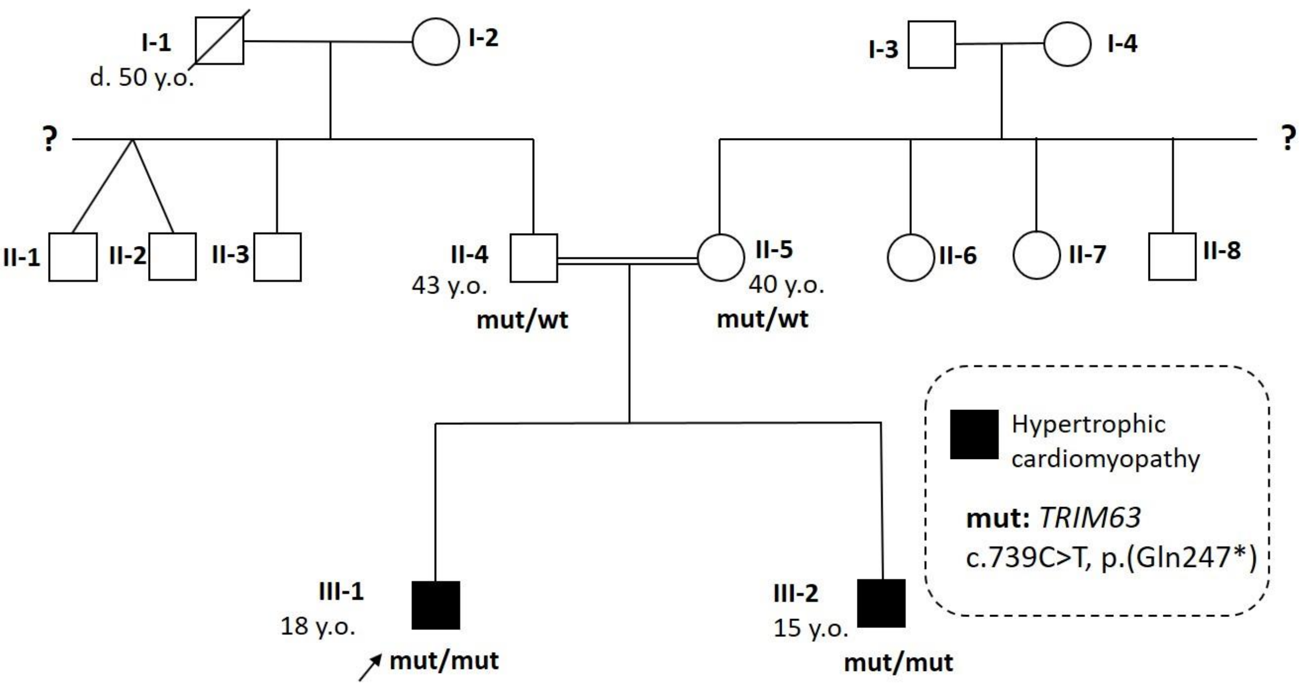

References

1. Tobita, T.; Nomura, S.; Morita, H.; Ko, T.; Fujita, T.; Toko, H.; Uto, K.; Hagiwara, N.; Aburatani, H.; Komuro, I. Identification of MYLK3 mutations in familial dilated cardiomyopathy. *Sci Rep* **2017**, *7*, 17495, doi:10.1038/s41598-017-17769-1.
2. Jokela, M.; Baumann, P.; Huovinen, S.; Penttila, S.; Udd, B. Homozygous Nonsense Mutation p.Q274X in TRIM63 (MuRF1) in a Patient with Mild Skeletal Myopathy and Cardiac Hypertrophy. *J Neuromuscul Dis* **2019**, *6*, 143-146, doi:10.3233/JND-180350.
3. Olive, M.; Abdul-Hussein, S.; Oldfors, A.; Gonzalez-Costello, J.; van der Ven, P.F.; Furst, D.O.; Gonzalez, L.; Moreno, D.; Torrejon-Escribano, B.; Alio, J., et al. New cardiac and skeletal protein aggregate myopathy associated with combined MuRF1 and MuRF3 mutations. *Hum Mol Genet* **2015**, *24*, 3638-3650, doi:10.1093/hmg/ddv108.
4. Lefta, M.; Campbell, K.S.; Feng, H.Z.; Jin, J.P.; Esser, K.A. Development of dilated cardiomyopathy in Bmal1-deficient mice. *Am J Physiol Heart Circ Physiol* **2012**, *303*, H475-485, doi:10.1152/ajpheart.00238.2012.
5. Marston, S.; Montgiraud, C.; Munster, A.B.; Copeland, O.; Choi, O.; Dos Remedios, C.; Messer, A.E.; Ehler, E.; Knoll, R. OBSCN Mutations Associated with Dilated Cardiomyopathy and Haploinsufficiency. *PLoS One* **2015**, *10*, e0138568, doi:10.1371/journal.pone.0138568.
6. Siegert, R.; Perrot, A.; Keller, S.; Behlke, J.; Michalewska-Wludarczyk, A.; Wycisk, A.; Tendera, M.; Morano, I.; Ozcelik, C. A myomesin mutation associated with hypertrophic cardiomyopathy deteriorates dimerisation properties. *Biochem Biophys Res Commun* **2011**, *405*, 473-479, doi:10.1016/j.bbrc.2011.01.056.
7. Liu, J.; Kong, X.; Lee, Y.M.; Zhang, M.K.; Guo, L.Y.; Lin, Y.; Lim, T.K.; Lin, Q.; Xu, X.Q. Stk38 Modulates Rbm24 Protein Stability to Regulate Sarcomere Assembly in Cardiomyocytes. *Sci Rep* **2017**, *7*, 44870, doi:10.1038/srep44870.
8. Ishikawa, T.; Sato, A.; Marcou, C.A.; Tester, D.J.; Ackerman, M.J.; Crotti, L.; Schwartz, P.J.; On, Y.K.; Park, J.E.; Nakamura, K., et al. A novel disease gene for Brugada syndrome: sarcolemmal membrane-associated protein gene mutations impair intracellular trafficking of hNav1.5. *Circ Arrhythm Electrophysiol* **2012**, *5*, 1098-1107, doi:10.1161/CIRCEP.111.969972.
9. Richards, S.; Aziz, N.; Bale, S.; Bick, D.; Das, S.; Gastier-Foster, J.; Grody, W.W.; Hegde, M.; Lyon, E.; Spector, E., et al. Standards and guidelines for the interpretation of sequence variants: a joint consensus recommendation of the American College of Medical Genetics and Genomics and the Association for Molecular Pathology. *Genet Med* **2015**, *17*, 405-424, doi:10.1038/gim.2015.30.
